# Genetic and Molecular Characterization of Submergence Response Identifies *Subtol6* as a Major Submergence Tolerance Locus in Maize

**DOI:** 10.1371/journal.pone.0120385

**Published:** 2015-03-25

**Authors:** Malachy T. Campbell, Christopher A. Proctor, Yongchao Dou, Aaron J. Schmitz, Piyaporn Phansak, Greg R. Kruger, Chi Zhang, Harkamal Walia

**Affiliations:** 1 University of Nebraska-Lincoln, Department of Agronomy and Horticulture, Lincoln, Nebraska, United States of America; 2 University of Nebraska-Lincoln, School of Biological Sciences, Lincoln, Nebraska, United States of America; 3 Nakhon Phanom University, Muang District, Thailand; China Agricultural University, CHINA

## Abstract

Maize is highly sensitive to short term flooding and submergence. Early season flooding reduces germination, survival and growth rate of maize seedlings. We aimed to discover genetic variation for submergence tolerance in maize and elucidate the genetic basis of submergence tolerance through transcriptional profiling and linkage analysis of contrasting genotypes. A diverse set of maize nested association mapping (NAM) founder lines were screened, and two highly tolerant (Mo18W and M162W) and sensitive (B97 and B73) genotypes were identified. Tolerant lines exhibited delayed senescence and lower oxidative stress levels compared to sensitive lines. Transcriptome analysis was performed on these inbreds to provide genome level insights into the molecular responses to submergence. Tolerant lines had higher transcript abundance of several fermentation-related genes and an unannotated *Pyrophosphate-Dependent Fructose-6-Phosphate 1-Phosphotransferase* gene during submergence. A coexpression network enriched for *CBF* (*C-REPEAT/DRE BINDING FACTOR*: *C-REPEAT/DRE BINDING FACTOR*) genes, was induced by submergence in all four inbreds, but was more activated in the tolerant Mo18W. A recombinant inbred line (RIL) population derived from Mo18W and B73 was screened for submergence tolerance. A major QTL named *Subtol6* was mapped to chromosome 6 that explains 22% of the phenotypic variation within the RIL population. We identified two candidate genes (*HEMOGLOBIN2* and *RAV1*) underlying *Subtol6* based on contrasting expression patterns observed in B73 and Mo18W. Sources of tolerance identified in this study (*Subtol6*) can be useful to increase survival rate during flooding events that are predicted to increase in frequency with climate change.

## Introduction

The frequency and intensity of extreme precipitation events (drought and flooding) are predicted to increase due to changes in climatic patterns. These changes in precipitation patterns will impact many maize-producing regions of the world, which receive considerable rainfall during early growing season [1, 2]. Maize is highly sensitive to waterlogging and submergence (complete inundation) during germination and seedling stages and leads to poor seedling establishment, stunted growth, and delayed development. Most of the previous research has focused on waterlogging, while submergence tolerance remains less characterized in maize [3–10].

At a cellular level, hypoxic conditions during submergence reduce protein translation and induce selectively translated proteins involved in glycolysis and the fermentative pathways [11]. The shift to anaerobic respiration allows the plants to continue producing ATP, albeit at a considerably lower rate, while carbon reserves are still available [12–14]. As part of a metabolic adaptation to hypoxia, several ATP consuming biochemical pathways are inhibited [15]. Furthermore, survival through prolonged inundation hypoxia also involves the use of inorganic pyrophosphate (PPi) as an alternative energy source and induction of enzymes that reduce reactive oxygen species (ROS) or cytoplasmic acidosis [12–14, 16, 17].

The molecular and physiological mechanisms involved in the submergence responses in plants have largely been characterized in two tolerant species, rice (*Oryza sativa*) and *Rumex palustris* [18–23]. One of the early responses to submergence involves the differential regulation of a suite of transcription factors belonging to the ethylene response factor (ERFs) gene family [20, 21, 24–28]. Rice *SUB1A*, a member of the ERF gene family, dramatically improves submergence tolerance in controlled and field conditions. *SUB1A* limits shoot elongation during submergence by repressing gibberellic acid (GA) levels and modulating GA signaling [20–22]. In striking contrast, two ERFs, *SNORKEL1* and *SNORKEL2*, confer submergence adaptation in deepwater rice by inducing rapid internode elongation through enhanced GA responses [25].

Given the economic significance of submergence tolerance for maize, our current knowledge of the submergence responses in maize is limited when compared to rice and *Rumex palustris*. The extent of genetic variation for submergence tolerance in maize is largely unexplored. Utilizing the natural variation in maize for submergence tolerance studies has become more tractable due to availability of genetic resources such the Nested Association Mapping (NAM) populations and high-density marker information [29]. The aims of this study were to (i) assess the natural variation for submergence tolerance in maize germplasm by screening the diverse maize founder lines, (ii) examine the molecular responses to submergence through transcriptional profiling of a subset of tolerant and sensitive inbreds, and (iii) elucidate the genetic basis of submergence tolerance through QTL analysis. We show that substantial variation for submergence tolerance exists in maize NAM population germplasm, and identify a major QTL associated with submergence tolerance.

## Results

### Maize inbreds exhibit significant phenotypic diversity for submergence tolerance

**Fig 1:**
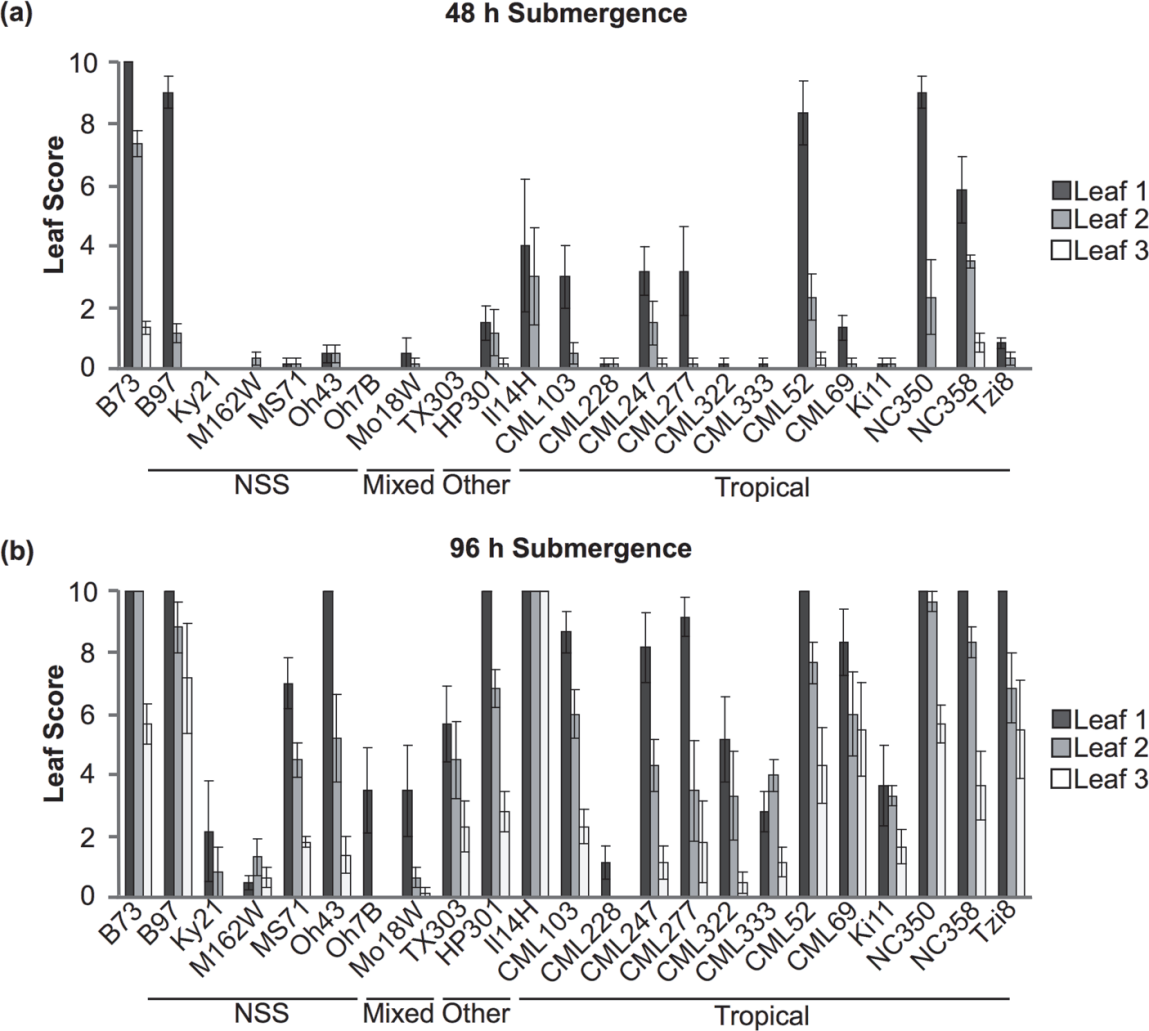
Phenotyping of NAM parents after 48 h and 96 h of complete inundation. Plants were completely submerged for 48 h (a) and 96 h (b) and scored daily for 7 d after removing from submergence. Plants were scored on a 0 to10 scale with a score of 0 showing no visual damage and 10 indicating complete senescence. Graphed are the scores of leaves 1 to 3 (1: oldest leaf, 3: youngest leaf) at 7 d. Genotypes with no senescence or other damage from submergence (e.g. Ky21 at 48 h) do not have a bar graph representation. Genotypes in which the leaf received the same score in each replicate have no error bars. Bars represent standard error, where n = 9 for this experiment. Genotypes were separated into subfamilies based on the classification of McMullen et al. [29]. NSS: non-stiff stalk.

A panel of 23 genetically diverse maize Nested Association Mapping (NAM) parental lines, representing five subpopulations, and B73 were screened for submergence tolerance for a period of 48 h or 96 h. The NAM founder lines were selected to represent maize diversity and include more than 50% tropical lines and 9 temperate lines [29]. Submerged plants were scored for visual damage using a scoring system specifically developed for this study (see Material and Methods section; S1 Fig and S2 Fig). A parallel set of non-submerged plants was maintained as controls. The scoring range was set from 0–10 and lower scores correspond to higher tolerance based on visual phenotyping. Based on the submergence tolerance scoring system, we observed considerable variation among maize NAM parents (Fig. 1). Our visual scoring of leaf 1 (oldest leaf), 2 and 3 (youngest leaf), identified several genotypes (B73, B97, CML52, Il14H, NC350 and NC358) with high sensitivity after 48 h of submergence. In contrast, at least 12 of the NAM parents had minimal or no visual damage from 48 h of submergence followed by 7 d recovery period (Fig. 1A). Scores based on 96 h of submergence, followed by 7 d recovery period were more reliable in identifying the highly tolerant inbreds. The identified tolerant lines (CML228, KY21, M162W and Mo18W) exhibited no or minimal signs of stress after 48 h and 96 h of submergence in the three replicates (Fig. 1). The four tolerant genotypes tested had either minor or no chlorosis on the lower leaves, while newer leaves remained turgid and green after 48 h and 96 h of submergence (S3 Fig). These results indicate that most maize inbreds are highly sensitive to even short duration of submergence.

### Submergence tolerant genotypes maintain lower reactive oxygen species levels

**Fig 2:**
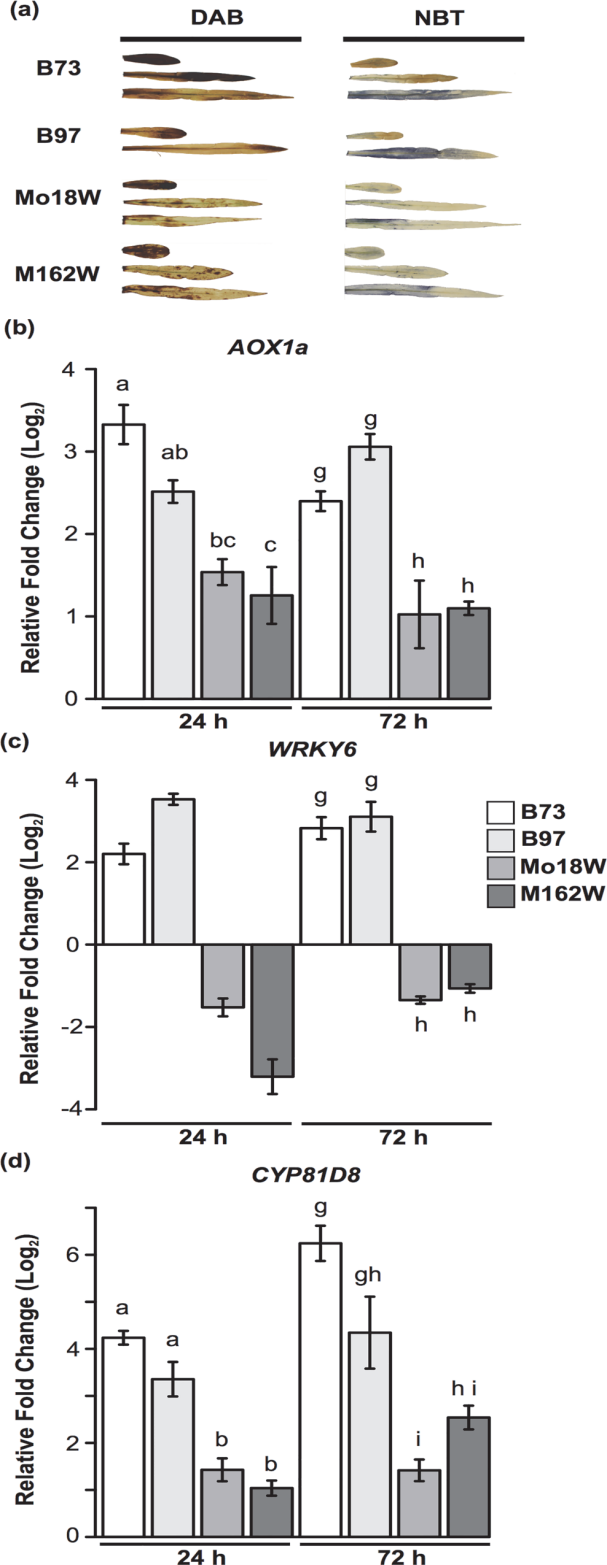
Detection of ROS and expression of ROS marker genes in inbreds with contrasting levels of submergence tolerance. (a.) Detection of H_2_O_2_ and superoxide in selected submergence tolerant and sensitive maize genotypes. A subset of four nested association mapping panel parents were selected based on visual leaf scoring for ROS staining assays after submergence. Hydrogen peroxide was detected using stain 3,3′-diaminobenzidine tetrahydrochloride (DAB), while superoxide was detected using nitroblue tetrazolium (NBT). Both assays were performed after 96 h of submergence. In B97, the third leaf was barely emerged and not used for the assay. (b-d) Real-time quantitative PCR of ROS genes. ROS genes were selected based on the study by Gadjev, Vanderauwera and Gechev [30] and showed a significant induction in response to at least two stresses that induce ROS formation in Arabidopsis. (b.) *ALTERNATIVE OXIDASE 1a* (*AOX1a*); (c.) *WRKY6* (d.) *CYTOCHROME P81 D8* (*CYP81D8*). All sample were collected at 24 h and 72 h after submergence. Expression levels are relative to shoot tissue of control plants at 24 h. Letters above bars indicate nonsignificant differences in expression determined using Tukey’s HSD (*p* < 0.05). Statistical tests were performed separately for each time point. Plots without letters (ie 2c 24 h) indicate that all comparisons are significantly different. Error bars display standard error where *n* = 3.

Because stress-associated senescence and loss of turgor in the sensitive genotypes are typical symptoms of disrupted cellular processes, we selected two tolerant (Mo18W and M162W) and two highly sensitive genotypes (B73 and B97) for estimating reactive oxygen species (ROS) levels in submerged plants (Fig. 2A). Based on Nitro Blue Tetrazolium (NBT) and Diaminobenzidine tetrahydrochloride (DAB) staining assays, which measure superoxide and hydrogen peroxide respectively, we observed that tolerant genotypes generally had lower levels of H_2_O_2_ and superoxide in leaves after 96 h of complete submergence compared to sensitive genotypes (Fig. 2A). B73 and B97 showed more intense DAB and NBT staining after 96 h of submergence suggesting that higher levels of superoxide and hydrogen peroxide were produced in the leaves. The lower ROS levels in submergence tolerant inbreds could be due to lower ROS production and/or more effective ROS scavenging.

To validate the results of NBT and DAB staining assays, we examined the expression of known ROS marker genes in the four inbreds after 24 and 72 h of submergence using quantitative real-time PCR (qPCR). The ROS marker genes were from derived from an Arabidopsis dataset for four oxidative stress treatments (methyl viologen, *Alternaria alternata* toxin, 3-aminotriazole, and ozone) [30]. Genes were selected if they showed an induction in at least two of the four stresses and had corresponding maize orthologs. The relative gene expression was measured using real-time quantitative PCR (qPCR) for *ALTERNATIVE OXIDASE 1a* (*AOX1a*; GRMZM2G125669). Both sensitive inbreds displayed a considerably higher induction of *AOX1a* in response to submergence when compared to M162W at 24h (Fig. 2B; *p* < 0.05). While at 72 h after submergence, the expression of *AOX1a* was significantly higher in both B73 and B97 compared to tolerant inbreds (Fig. 2B; *p* < 0.05). Maize *WRKY6* ortholog (GRMZM5G871347) showed contrasting expression patterns in response to submergence in maize (Fig. 2C; *p* < 0.05). The expression of *WRKY6* was induced in both B73 and B97 by more than two-fold at 24 and 72 h after submergence relative to control samples, while in tolerant inbreds the expression of *WRKY6* was down-regulated at both time points relative to controls. A third marker gene, *CYP81D8* (GRMZM2G087875) was expressed at significantly lower levels in both tolerant lines at 24 h after submergence compared to sensitive lines (Fig. 2D; *p* < 0.05). While at 72 h after submergence, *CYP81D1* showed significantly lower expression in Mo18W when compared to both sensitive inbreds. Collectively, the staining assays and quantitative expression analysis suggest that the relatively lower survival and poor recovery of the sensitive inbreds could partly be due to higher ROS levels and downstream transcriptome changes that accompany oxidative stresses.

### Sensitive and tolerant maize inbreds have distinct transcriptome dynamics during submergence

We performed RNAseq analysis using shoot tissue sampled at 24 and 72 h after submergence for the sensitive genotypes (B73 and B97) and tolerant genotypes (M162W and Mo18W). Non-submerged shoot samples were collected at 24 h as controls. We sampled at 24 h to capture the early responses to short-term submergence. Because, by 96 h, the tolerant genotypes were clearly distinguishable from the sensitive genotypes based on ROS staining assays, we reasoned that the 72 h time point could provide insights on the inducible tolerance responses before the visual phenotypes appear. The complete set of differentially expressed genes is provided as S1 File (FDR < 0.001).

Submergence resulted in a major shift in the transcriptome of all four maize genotypes as indicated by a large number of responsive genes (Table 1). Two numeric features emerged from submergence responsive differential expression analysis. The tolerant M162W had almost half the number of differentially expressed genes at 24 h relative to the other genotypes. The three replicates for M162W had a high Spearman correlation (*R* ^*2*^ = 0.95–0.98) suggesting that a smaller set of differentially expressed genes was not due to variation among the three biological replicates. The number of differentially expressed genes in M162W at 72 h was comparable to other genotypes. These results suggested that short-term submergence had a delayed effect on M162W transcriptome relative to other genotypes.

**Table 1:** Summary of differential expression analysis.

	B73		B97		Mo18W		M162W		Commonly Expressed in Sensitive Lines		Commonly Expressed in Tolerant Lines	
	Up	Down	Up	Down	Up	Down	Up	Down	Up	Down	Up	Down
**24 h**	3603	3018	2390	2168	2292	2123	1344	575	1969	1304	1041	425
**72 h**	3389	2262	2439	1816	3222	3068	3490	2801	1820	1096	1899	1745

Number of differentially expressed transcripts from comparisons between submerged and control plants. All transcripts showed significant differences in expression (FDR <0.001).

Despite the phenotypic similarities observed in the tolerant lines, very few genes displayed similar expression patterns after 24 h of submergence (S4 Fig). In contrast, the transcriptional response of the sensitive lines was mostly shared. Approximately 50% of the genes that were differentially expressed in response to submergence in B73 at 24 h displayed similar expression patterns in B97 at 24 h after submergence (Table 1). The overlapping expression patterns observed in sensitive lines suggest that they may share a similar response to submergence.

### Tolerant lines exhibit higher expression of hypoxia-associated energy homeostasis genes

**Fig 3:**
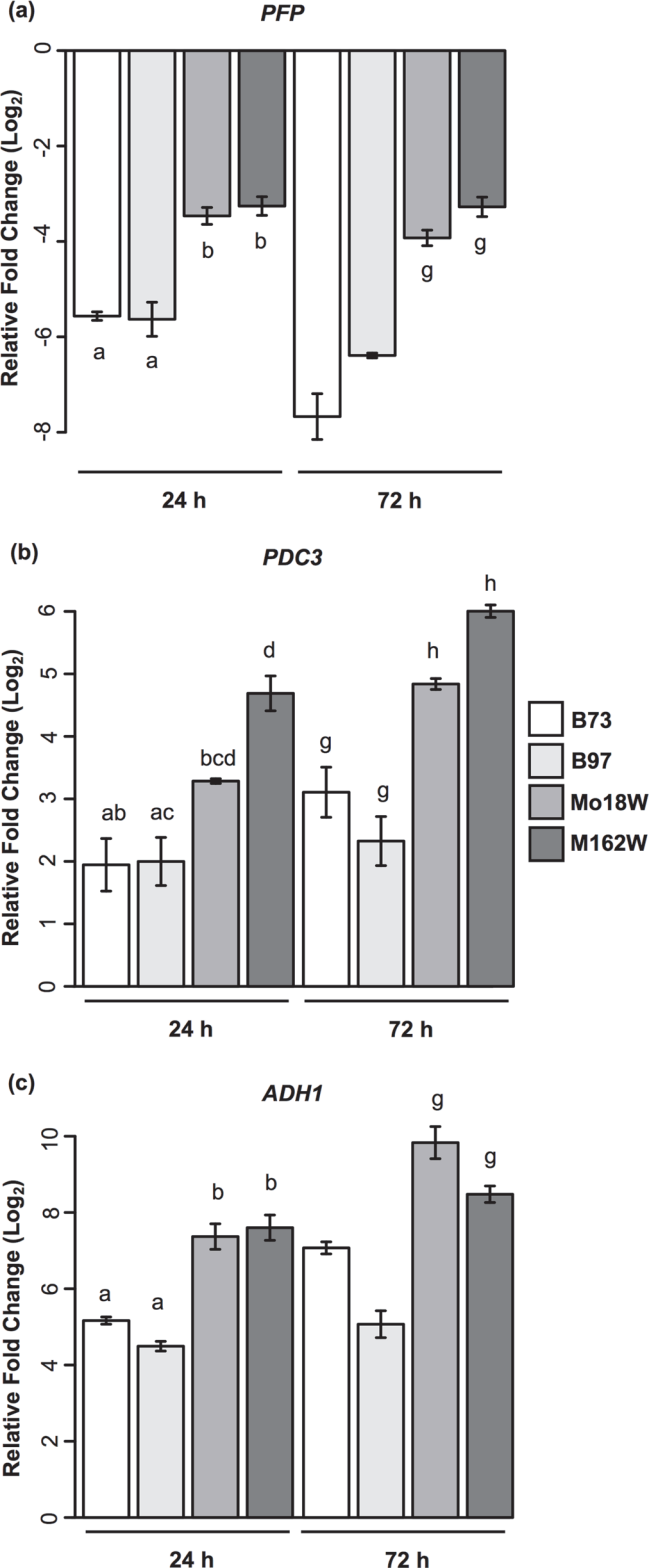
Real-time quantitative PCR of *PYROPHOSPHATE-DEPENDANT FRUCTOSE-6-PHOSPHATE 1-PHOSPHOTRANSERASE* (*PFP*) and anaerobic glycolysis genes during submergence. (a.) The expression of an unannotated *PYROPHOSPHATE-DEPENDANT FRUCTOSE-6-PHOSPHATE 1-PHOSPHOTRANSERASE* (*PFP*) gene in submerged shoot tissue during submergence. (b.) *PYRUVATE DECARBOXYLASE3* (*PDC3*) expression in shoot tissues during submergence. (c.) *ALCOHOL DEHYDROGENASE1* (*ADH1*) transcript levels in submerged shoot tissue. All sample were collected at 24 h and 72 h after submergence. Expression levels are relative to shoot tissue of control plants at 24 h. Letters above bars indicate nonsignificant differences in expression determined using Tukey’s HSD (*p* < 0.05). Statistical tests were performed separately for each timepoint. Plots without letters (ie 3a 72 h and 3c 72 h) indicate that all comparisons are significantly different. Error bars display standard error where *n* = 3.

We explored the possibility that unannotated genes may be contributing to submergence response in maize (S2 File). A notable finding from this analysis was a transcript (TCONS_00005048) with similarity to the alpha subunit of *PYROPHOSPHATE-DEPENDENT FRUCTOSE-6-PHOSPHATE 1-PHOSPHOTRANSFERASE (PFP)* protein in maize. Although the expression of *PFP* was strongly reduced by submergence in all lines, the transcript abundance of *PFP* was higher in the tolerant lines during submergence due to a relatively gradual decline compared to the rapid drop in transcript levels observed in the sensitive inbreds. The expression of this *PFP* transcript was confirmed using qPCR, indicating that it is a bona fide transcript (Fig. 3A). Several other genes participating in ethanolic fermentation downstream of *PFP* also had higher transcript abundance in the tolerant lines after 24 and 72 h of submergence relative to controls. For instance, a *PYRUVATE DECARBOXYLASE3* gene (*PDC3*; GRMZM2G087186) showed more than a 3 and 4-fold induction in M162W and Mo18W, respectively after 24 h of submergence when compared to control plants (Fig. 3B). The transcript abundance of *PDC3* continued to increase significantly in tolerant lines during submergence, showing a 4.8 and 6-fold induction in Mo18W and M162W, respectively after 72 h of submergence. A similar trend in *PDC3* expression was also observed in B73 during submergence; however, the expression in response to submergence was considerably lower relative to the tolerant lines. A gene encoding *ALCOHOL DEHYDROGENASE1* (*ADH1*; GRMZM2G442658) showed more than a seven-fold induction in Mo18W and M162W after 24 h of submergence (Fig. 3C). The expression increased further after 72 h of submergence, with more than a 9.5 and eight-fold up-regulation observed in Mo18W and M162W, respectively. Although *ADH1* was induced by submergence in the sensitive lines, the induction was considerably lower relative to the tolerant lines.

### Oxidative stress associated gene families are differentially regulated during submergence in maize

To identify pathways that may be affected during submergence, gene ontology (GO) enrichment analysis was conducted on differentially expressed gene lists (S4 File). Transcripts associated with protein phosphorylation and ubiquination, were enriched among up-regulated genes in all in accessions at 24 and 72 h after submergence (S4 File). Several notable features were observed in genes that showed similar expression patterns in both tolerant and sensitive lines. Transcripts associated with trehalose metabolism were enriched among up-regulated transcripts in Mo18W at 24 and 72 h after submergence, suggesting that this may be a unique component of the transcriptional response to submergence in this inbred (S4 File; GO:0005991, GO:0005992). Genes involved in apoptosis and cell death were overrepresented (*p* < 0.05) in up-regulated genes in sensitive lines, suggesting that processes associated with senescence were triggered as part of an early response to submergence in these accessions (S5C Fig; GO:0006915, GO:0012501, GO:0008219, GO:0016265). Several notable gene families with role in senescence were down-regulated in the tolerant lines when compared to one or more sensitive line during submergence. Redox homeostasis related transcripts, such as *Gluathione S-Transferase* (*GST*) and *Alternative Oxidase* genes (*AOX*) were expressed at significantly higher levels in sensitive lines compared to tolerant varieties (S6A Fig). Both GST and *AOX* are critical for redox homeostasis. *AOX* competes with cytochrome bc1 complex in mitochondria, reducing the production of superoxide radicals [31, 32].

We performed transcription factor enrichment analysis and found significant enrichment for several transcription factor families such as *ERF*, *NAC* (*NAM*, *ATAF*, and *CUC*) and *WRKY* across genotypes in response to submergence (*p*<0.05; Table 2; S1 Table). Although these transcription factor families are involved in many diverse processes, several orthologous genes known to be involved with oxidative stress responses and ethylene signaling were expressed at significantly higher levels in sensitive inbreds relative to tolerant lines. Besides the genes that respond to submergence, genotypic comparisons showed contrasting expression patterns between the tolerant and sensitive lines for these transcription factor families (Table 3; S2 Table). We observed that the *NAC* family transcription factors were significantly enriched among down-regulated genes in all comparisons between tolerant and sensitive lines, except comparisons between Mo18W and B97 (p <0.05; Table 3). A gene similar to *AtNAC029*, which is induced during leaf senescence in Arabidopsis, displayed considerably lower expression in all tolerant lines when compared to sensitive inbreds at both 24 and 72 h after submergence ([33]; S6B Fig). Furthermore, SUPPRESSOR *OF GAMMA RADIATION 1* (*SOG1*) (GRMZM2G078954) in Arabidopsis showed considerably lower levels of expression in Mo18W when compared to both sensitive lines at 24 and 72h after submergence. In Arabidopsis, *SOG1* is a central player in DNA damage response, inducing hundreds of genes involved in DNA repair [34, 35].

**Table 2:** Transcription factor enrichment analysis from comparisons between treatments.

		24 h			72 h		
		*AP2-ERF*	*NAC*	*WRKY*	*AP2-ERF*	*NAC*	*WRKY*
**B73**	**Down**	4.0%	1.9%	3.8%	3.3%	1.3%	3.8%
	**Up**	13.9%***	15.2% ***	12.5% **	13.1% ***	17.1% ***	13.1% **
**B97**	**Down**	2.2%	1.3%	1.3%	2.2%	0.6%	1.3%
	**Up**	10.6% ***	10.1% ***	11.3% ***	9.9% ***	12.7% ***	9.4% **
**Mo18W**	**Down**	2.2%	1.3%	2.5%	4.7%	5.1%	4.4%
	**Up**	10.6% ***	10.8% ***	8.8% *	11.3% ***	13.9% ***	7.5%
**M162W**	**Down**	0.7%	0.0%	3.1% *	4.4%	1.9%	3.1%
	**Up**	10.9%***	5.1% ***	3.8%	14.6% ***	16.5% ***	14.4% ***

Subset of enriched transcription factors that were significantly enriched among differentially expressed genes. Differentially expressed genes were identified from comparisons between submerged and control plants at 24 and 72 h (FDR < 0.001). Asterisks denote statistical significance as determined using hypergeometric test: * p≤0.05, ** p≤0.01 and *** p≤0.001. *NAC*: *NAM*, *ATAF*, *CUC1*.

**Table 3:** Transcription factor enrichment analysis from comparisons between genotypes. Subset of enriched transcription factor identified from comparisons between genotypes at 24 and 72 h after submergence. Differentially expressed genes were identified from comparisons between submerged and control plants at 24 and 72 h (FDR < 0.001). Asterisks denote statistical significance as determined using hypergeometric test: * *p*≤0.05, ** *p*≤0.01 and *** *p*≤0.001. *NAC*: *NAM*, *ATAF*, *CUC1*.

		24 h			72 h		
		*AP2-ERF*	*NAC*	*WRKY*	*AP2-ERF*	*NAC*	*WRKY*
**Mo18W vs B73**	**Down**	6.2%	8.2% *	5.6%	7.7%	11.4% **	13.1% ***
	**Up**	3.2% *	0.6%	1.3%	1.5%	0.6%	0.6%
**Mo18W vs B97**	**Down**	3.3%	5.1%	5.0%	3.6%	10.1% ***	12.5% ***
	**Up**	2.9%	1.3%	2.5%	1.8%	3.2%	1.3%
**M162W vs B73**	**Down**	5.1%	8.9% **	8.8% **	2.9%	5.1% *	1.9%
	**Up**	1.5%	0.0%	0.6%	3.3%	3.2%	2.5%
**M162W vs B97**	**Down**	3.6%	7.0% **	6.9% **	6.2% *	9.5% ***	3.8%
	**Up**	4.4%	1.3%	2.5%	2.2%	3.8%	1.9%

*WRKYs* were enriched among the down-regulated genes in M162W when compared to both sensitive inbreds at 24 h after submergence. However, at 72 h a significant enrichment was observed among the down-regulated genes of Mo18W when compared to the sensitive lines (Table 3). One notable WRKY gene, which is similar to *WRKY53* (GRMZM2G063880) in Arabidopsis, showed considerable higher expression in both sensitive inbreds when compared to M162W and Mo18W at both 24h and 72h after submergence respectively (S6B Fig). In Arabidopsis, *WRKY53* has been shown to be a positive regulator of leaf senescence [36–38]. Two putative WRKY genes that are similar to *AtWRKY33* (GRMZM2G169966, GRMZM2G148087) showed lower expression in M162W at 24 and Mo18W at 72h when compared to both sensitive lines (S6B Fig). *WRKY33* is involved in ethylene-mediated thermotolerance and was proposed to regulate ethylene signaling via a positive feedback mechanism [39]. More recently it was shown that *WRKY33* regulates the expression of the ethylene biosynthesis gene, *ACS6* [40]. Collectively, our analysis of *WRKY* and *NAC* factors suggested that expression patterns of several of these genes, with orthologs known to be associated with senescence correlated with submergence sensitivity response. Surprisingly, some of these transcription factors and senescence-associated transcriptional signatures were triggered within 24 h of submergence in the sensitive maize genotypes.

### ERFs are an integral component of the early submergence response in maize

We found that ERFs were significantly enriched among up-regulated genes in response to submergence (*p* < 0.001). Eighteen ERFs were up-regulated in all four lines at 24 h after submergence, while at 72 h, 19 ERF genes were up-regulated in response to submergence (Table 2; S1 Table). We also found a notable ERF that was induced only in the tolerant lines at 24 h. The expression of maize ortholog of *REDOX-RESPONSIVE TRANSCRIPTION FACTOR* (*RRTF1*; GRMZM2G138396; S7A Fig) was induced by approximately 7- and 4.6-fold in Mo18W and M162W respectively. *RRTF1* is a key member of a regulatory network required for redox homeostasis in Arabidopsis and could play a role in ameliorating the redox imbalance associated with submergence in the tolerant inbreds [41].

### Induction of B-2 ERFs by submergence is conserved across species

Given the significant differential response of maize ERFs during submergence, we extended our expression analysis across multiple species for the maize submergence responsive ERFs. We mined the published submergence/hypoxia transcriptome datasets from Arabidopsis and rice for orthologous *ERFs* that were differentially regulated during maize submergence [22, 42–43] (GSE41103; GSE18930;, GSE24077). Results from the cross-species comparisons are summarized in S3 Table. One notable feature to emerge from the *ERF* family analysis was the expression of seven *ERF* gene family members corresponding to the *ERF* clade B-2 (Arabidopsis group VII *ERFs*; S7A Fig, S8 Fig; S5 File). These transcription factors were characterized by strong up-regulation at both time points in the four maize genotypes (Table 2; S1 Table). Arabidopsis and rice orthologs of this subgroup were also strongly induced in response to submergence, indicating a conserved response across these three species. The orthologs in this clade play important roles in hypoxia and submergence tolerance, and include the *HYPOXIA-RESPONSE ERFs* (*HRE1* and *HRE2*) in Arabidopsis, and the rice *SUB1A* gene [26, 27]. Collectively, our maize data and reports from Arabidopsis and rice suggest that the *ERF* gene family and specifically, the subgroup B-2 exhibited a conserved response to submergence across multiple species. While the transcriptional patterns of B-2 ERFs are conserved across species during submergence, members of the B-2 ERF subfamily have also been reported be regulated at the post-translational level [27, 28].

### CBF transcriptional network is submergence inducible in maize

**Fig 4:**
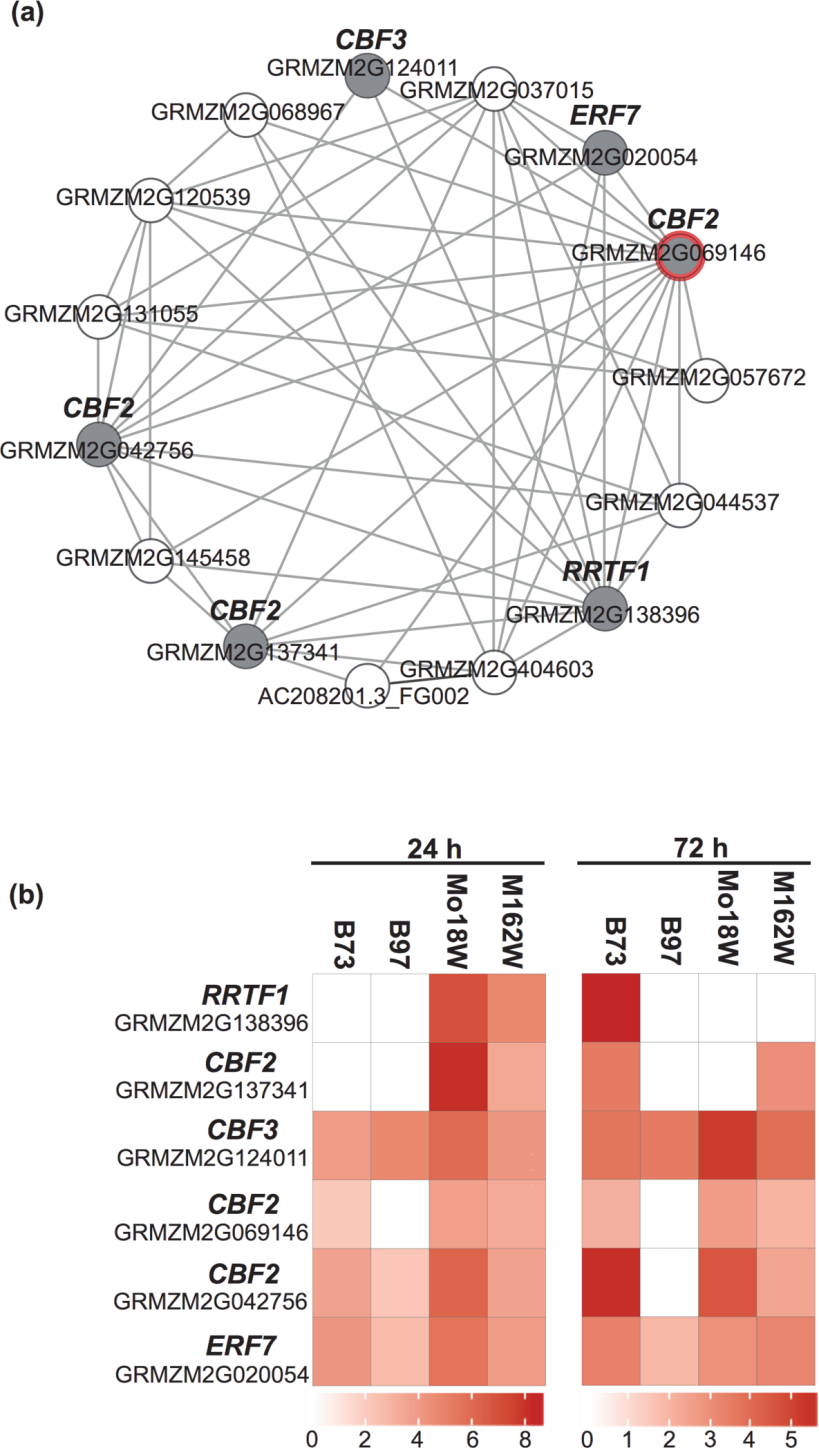
*CBF* (*C-REPEAT/DRE BINDING FACTOR*) co-expression cluster is induced by submergence in maize. (a.) *CBF* co-expression network generated form genes differentially expressed at 24 h in comparisons between submerged and control plants. Each gene in the network is represented as a circle. (b.) Genes highlighted in the heatmap are represented by grey circles, while that with a red outline indicates the seed gene for the network. The heatmap highlights the expression of a subset of genes in B73, B97, Mo18W and M162W after 24 h of submergence. (c.) Expression of highlighted genes at 72 h after submergence. All genes showed significant differences in expression between submerged and control plants (FDR <0.001). The scale indicates log_2_ fold-change. Up-regulation indicates higher expression in submerged samples. *CBF2*: *C-REPEAT/DRE BINDING FACTOR2*; *CBF3*: *C-REPEAT/DRE BINDING FACTOR3*; *ERF7*: *ETHYLENE RESPONSE FACTOR7*; *RRTF1*: *REDOX REPONSIVE TRANSCRIPTION FACTOR1*

We identified four submergence-responsive *ERF* genes that are orthologous to the Arabidopsis *C-REPEAT/DRE BINDING FACTOR2* (*CBF2*; At4g25470) and another *ERF* orthologous to *C-REPEAT/DRE BINDING FACTOR3* (*CBF3*; At4g25480). These *CBF* genes were up-regulated at almost all time points in the four genotypes indicating that the maize *CBF* pathway was triggered by submergence. Strikingly, the induction of *CBF2* genes (GRMZM2G069146 and GRMZM2G069126) was higher in the tolerant Mo18W compared to other genotypes after 24 and 72 h of submergence. Arabidopsis *CBF2* gene is known to regulate a suite of downstream genes. To identify genes that are co-expressed with the two maize *CBF2* orthologs, we utilized the Maize Coexpression Browser (COB v1.02; http://csbio.cs.umn.edu/COB/), which generates co-expression networks from a maize developmental atlas dataset. Using the *CBF2* orthologs as seeds, two clusters were generated consisting of 23 and 17 genes from GRMZM2G069146 and GRMZM2G069126, respectively (Fig. 4A; S9 Fig). Approximately 61% of the genes (14 of 23) populating the GRMZM2G069146 network showed significant expression differences between control and submerged plants after 24 h (Fig. 4B). While in the GRMZM2G069126 cluster only 4 of 17 genes showed significant differences between control and submerged samples for at least one genotype. Notably, half of these genes in the larger GRMZM2G069146 cluster corresponded to *ERFs*, four of which encoded putative *CBF* genes. The presence of multiple *CBF* genes in this cluster is not surprising, considering that many *CBF* genes are co-expressed and regulate different aspects of the cold response in Arabidopsis [44, 45]. However, higher up-regulation of these genes in Mo18W suggested that *CBFs* could be an important component of the submergence tolerance response in this particular inbred.

### Subtol6, a major QTL on chromosome 6 is associated with submergence tolerance

**Fig 5:**
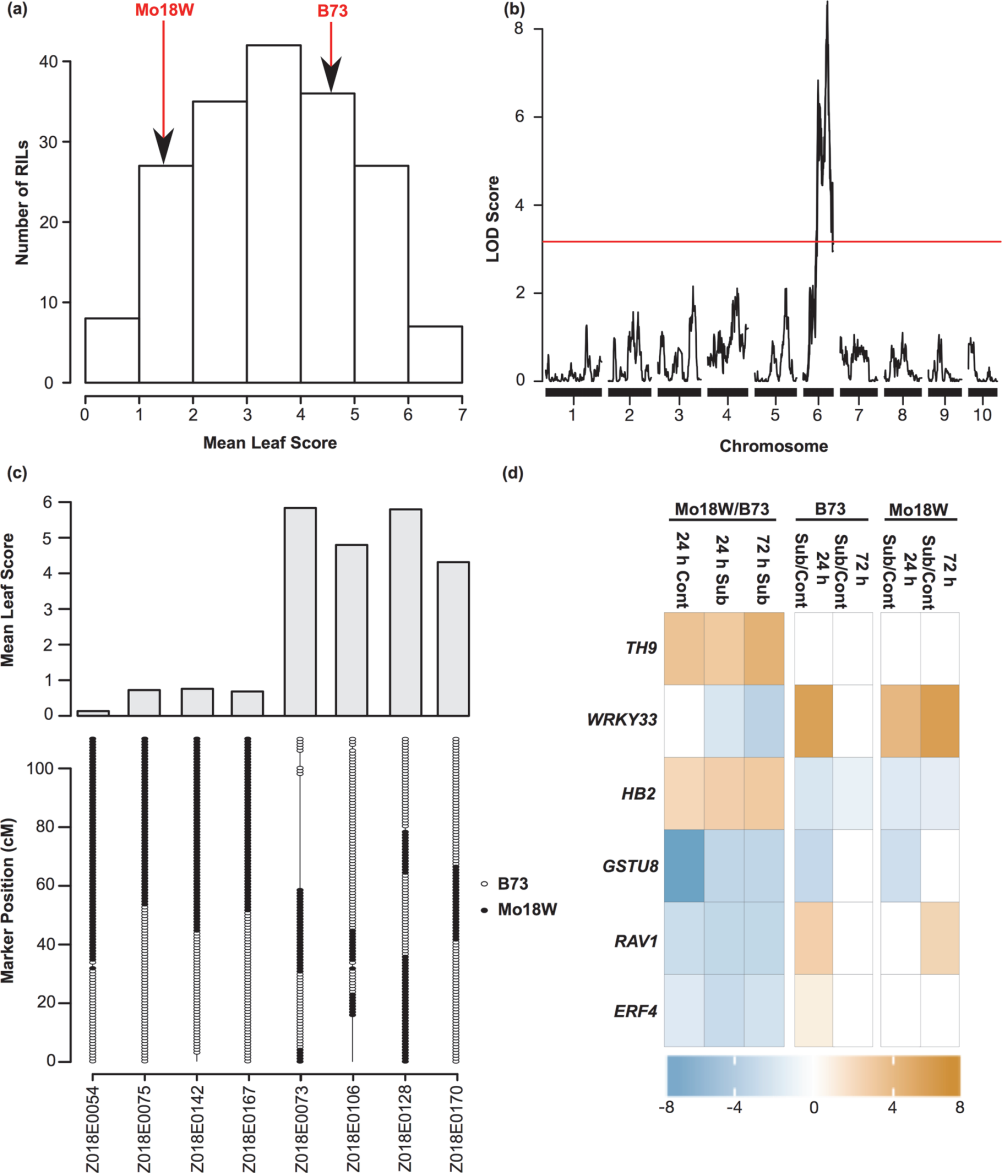
*Subtol6* is associated with submergence tolerance in maize. (a.) Distribution of mean leaf scores for Mo18W x B73 RIL mapping population and parental inbreds. Leaf score was averaged across leaves 1 to 3. Scores for parental inbreds are indicated with red arrows. (b.) Interval mapping results for QTL identified in the Mo18W x B73 RIL family from the NAM population. LOD threshold was determined using 10,000 permutations. (c.) Observed genotypes of chromosome 6 for a subset of tolerant and sensitive RILs. Markers with the B73 allele are represented by open ovals, while Mo18W alleles are represented by closed ovals. (d.) Expression of a subset of genes that fall within the Bayes credible interval for the most significant marker. Expression data was derived from RNA sequencing of B73 and Mo18W after 24 h and 72 h after submergence. All genes between ∼141.4 to 162.8 Mb on chromosome 6 and showing significant differences in expression in comparisons between B73 and Mo18W were considered (FDR <0.001). The scale indicates log_2_ fold-change. Up-regulation indicates higher expression Mo18W relative to B73. *TH9*: *THIOREDOXIN9* (GRMZM2G701204); *WRKY33* (GRMZM2G169966); *HB2*: *HEMOGLOBIN2* (GRMZM2G168898); *GSTU8*: *GLUTATHIONE S-TRANSFERASE8* (GRMZM2G097989); *RAV1*: *RELATED TO ABI3/VP1 1* (GRMZM2G059939); *ERF4*: *ETHYLENE RESPONSE FACTOR4* (GRMZM2G020150)

We selected the RIL family derived from the cross between the tolerant Mo18W and sensitive B73 lines in the NAM population to study the genetic basis of submergence tolerance in Mo18W. To this end we screened 166 RILs for submergence tolerance using the protocol described earlier for screening the founder lines (Fig. 5A) [29]. QTL analysis was performed with a publically available set of 1478 markers [46]. A major QTL, that we named *Submergence Tolerance 6* (*Subtol6)* was detected on chromosome 6. The *Subtol6* region spans ∼ 20 Mb (141–162 Mb) with the most significant marker located at ∼162 Mb (Fig. 5B). This QTL explains 22% of the total phenotypic variation for mean leaf score. To determine whether *Subtol6* is associated with submergence tolerance or sensitivity, we examined the mean leaf score for RILs harboring the Mo18W and B73 alleles at this location. RILs harboring the Mo18W allele for *Subtol6* were more tolerant than RILs with the B73 allele, suggesting that this region from Mo18W contains alleles conferring submergence tolerance in Mo18W relative to B73 (Fig. 5C).

To identify candidate genes for submergence tolerance in the *Subtol6* region, we mined the submergence transcriptome data for B73 and Mo18W from our RNAseq analysis. All genes falling within the 95% Bayes credible interval around the most significant marker (141–162 Mb on chromosome 6) were considered for expression analysis. Sixty-seven genes within this region showed significant differences (FDR < 0.001) in transcript abundance between Mo18W and B73 under control, or after 24 and 72 h of submergence (S3 File). Six of these differentially expressed genes underlying the *Subtol6* region have been previously reported to be involved in abiotic stress responses, hypoxia and senescence/oxidative stress (Fig. 5D). Two notable candidate genes are *RELATED TO ABA-INSENSITIVE3(ABI3)/VIVIPAROUS1* (*RAV1;* GRMZM2G059939) and *HEMOGLOBIN2* (*HB2;* GRMZM2G168898). *RAV1*, an *ERF* located at approximately 162.6 Mb, was expressed at significantly lower levels in Mo18W when compared to B73 in control and submerged conditions (S10A Fig). The position of *RAV1* is in close proximity to the most significant marker for *Subtol6* at ∼162 Mb. Arabidopsis *RAV1* is known to positively regulate leaf senescence [47]. The second candidate gene, *HEMOGLOBIN2* (*HB2*), which encodes for a non-symbiotic hemoglobin protein was identified at approximately 159.1 Mb on chromosome 6. *HB2* was expressed at higher level in Mo18W relative to B73 in both control and submerged conditions (S10B Fig). Hemoglobin proteins in maize have been associated with repressing ROS levels and maintaining energy status of maize cells during hypoxic conditions [48–51].

## Discussion

We have characterized the genetic variation for submergence tolerance in the maize NAM parent diversity panel and identified two inbreds with significantly higher submergence tolerance during the early seedling stage. These results have provided a means to map and characterize a major submergence tolerance QTL (*Subtol6*) using RILs derived from Mo18W and B73. Interestingly, the RILs harboring the B73 allele at *Subtol6*, were considerably more sensitive to submergence than those with the Mo18W allele. Since B73 is widely used in many U.S. breeding programs, this suggests that there is a need for improving submergence tolerance in lines that have retained alleles associated with B73 submergence sensitivity.

The submergence tolerant lines identified from our experiments displayed delayed senescence in response to complete submergence. Lower ROS levels were observed in the tolerant lines during submergence. An increase in ROS has been reported in plants exposed to submergence and hypoxic conditions [52, 53]. One of the likely candidate genes that we propose for *Subtol6* is *HB2*. This is based on the expression differences between the parent inbreds (Mo18W and B73) and previously reported role of *HB2* in plant survival in low oxygen or low ATP conditions [48–51]. A recent report has linked lower *HB2* levels with the triggering of programmed cell death, mediated by higher ROS levels in maize embryogenic tissue [51]. Our observations that the tolerant Mo18W has higher *HB2* transcript abundance, lower ROS levels and minimal senescence after submergence stress present *HB2* as an attractive candidate for *Subtol6*.

The second candidate gene underlying the *Subtol6* that we propose is the maize ortholog of *AtRAV1*, a member of the ERF gene family. Maize *RAV1* transcript abundance was lower in Mo18W relative to B73 under control and submerged conditions. Although, *RAV1* expression is induced in both Mo18W and B73, the up-regulation in B73 is higher than Mo18W in response to submergence. The differential expression pattern of *RAV1* between Mo18W and B73 is noteworthy because overexpression of *RAV1* in Arabidopsis is known to lead to early senescence. In wildtype Arabidopsis plants, *RAV1* expression is induced during onset of senescence [47]. This is consistent with the increased senescence observed in B73 relative to Mo18W after submergence stress. Although our selection of candidates for *Subtol6* is based on gene expression level differences, it is possible that the genetic basis of the *Subtol6* gene(s) could be due to sequence polymorphism in the underlying gene that has comparable transcript abundance during submergence and in control conditions. It is also possible that Mo18W may have additional gene(s) not present in the reference genome based on B73 as was the case with the rice *Sub1* locus. Future characterization of the *Subtol6* locus will address these possibilities and elucidate the casual gene underlying *Subtol6*.

Several *CBF/DREB* genes were induced in response to submergence in maize. This response was only observed in maize and is missing in rice and Arabidopsis. *CBF* genes act together in a transcriptional network and are expressed in response to a variety of conditions such as low temperature, dehydration, mechanical agitation, and senescence [54]. Notably, the induction of the CBF transcriptional network was stronger in the tolerant Mo18W, indicating that a *CBF*-mediated response may be more active in this line. In our study, submergence-induced senescence was delayed considerably in Mo18W compared to B73 and B97. Studies by Sharabi-Schwager et al. have shown that the ectopic expression of *CBF* genes negatively regulates senescence in Arabidopsis [55, 56]. Although further studies are needed to determine the underlying cause of delayed senescence in Mo18W, the higher expression of the *CBF* transcriptional network highlights an interesting feature of the submergence response of this inbred and merits further exploration.

Although the tolerant lines have very different genetic backgrounds, at the molecular level, several interesting features were shared. Both tolerant inbreds had higher transcript abundance of ethanol fermentation related genes, specifically *PDC3* and *ADH1* compared to sensitive inbreds. In hypoxic conditions, PDC catalyzes the conversion of pyruvate to acetaldehyde, which is then converted to ethanol by ADH providing NAD^+^ for glycolysis [16]. An unannotated transcript showing homology to the alpha subunit of *PYROPHOSPHATE-DEPENDENT FRUCTOSE-6-PHOSPHATE 1-PHOSPHOTRANSFERASE (PFP)* protein in maize was relatively less repressed in both tolerant lines compared to sensitive lines during submergence. During anoxic conditions the levels of ATP declines dramatically, and pathways that consume ATP are replaced by those that utilize PPi [13, 14, 57, 58]. In rice seedlings, the activity of PFP increased during prolonged oxygen deprivation, and the activity of *PFP* was dependent on protein levels rather than protein kinetics [12]. Furthermore, higher glycolytic rates, and higher protein levels and activity of *PFP* were reported in anoxia tolerant rice [18]. Although the exact function of *PFP* in plants is not entirely clear, it is thought that fructose-6-phosphate could be phosphorylated by *PFP* rather than phosphofructose kinase during anoxia. This mechanism could increase the ATP yield of glycolysis by as much as 50% during anaerobiosis [12]. To date no studies have explored the importance of *PFP* in hypoxia/submergence tolerance in maize, however the expression pattern of *PFP* in tolerant genotypes in our study presents an intriguing possibility for a role in submergence tolerance in maize.

Complete submergence of maize during an early vegetative stage is an event that could contribute to substantial seedling leaf senescence with resultant yield losses. Precipitation events leading to maize submergence are likely to increase in frequency with the predicted climatic shifts in precipitation patterns. Despite the economic implications of this situation, very few studies have explored submergence tolerance in maize [7, 59]. Here, we have identified a major QTL for submergence tolerance and characterized the molecular responses of a diverse set of maize inbreds to submergence. Future work will focus on identification and functional characterization of the major *Subtol6* gene conferring submergence tolerance in Mo18W.

## Materials and Methods

### Post-submergence visual scoring of NAM parents

Initial phenotypic screening for submergence tolerance was conducted using twenty-three genetically diverse maize (*Zea mays*) inbreds from the Nested Association Mapping (NAM) parent panel, representing five subfamilies, and B73 [29]. The genotypes used in this study and their corresponding subfamilies are listed in S4 Table. Seeds of each genotype were sown directly in soil and grown in greenhouse conditions. Temperature was maintained between 26.7 to 32.2°C during the day and 26.1 to 29.4°C during the night. The relative humidity ranged from 35% to 70%. Supplemental lighting was supplied by 1000-watt metal halide bulbs (Philips Lighting Co., Somerset, NJ, USA) from 0700 to 1800 hrs. Light intensity was measured at approximately 1200 hrs one day prior to submergence with LI-250A light meter and ranged from approximately 378 ± 62.4 μmol m^−2^ s^−1^ (LI-COR Biosciences, Lincoln, NE, USA). Pots were irrigated twice a day with approximately 50 ml of water. Half-strength Hoagland solution (50 ml) was applied once the first true leaf was visible to ensure that plants had adequate nutrition throughout the duration of the experiment [60]. Uniform seedlings (3 seedlings per inbred) were transferred to 416 L tubs at the V2 stage (three leaf stage) for submergence treatment. The water in the tubs was allowed to reach near ambient room temperatures (24–27°C) 24 to 48 h prior to submerging the plants. Plants were arranged in a complete randomized design, and were arranged towards the center to avoid shading by the walls of the tub. Plants were scored using a visual scoring system for the duration of one week post-submergence (S1 Fig). During the recovery period the temperatures maintained between 26.7 to 32.2°C during the day and 26.1 to 29.4°, and the relative humidity ranged from 35% to 70%. Supplemental lighting was supplied by 1000-watt metal halide bulbs (Philips Lighting Co., Somerset, NJ, USA) from 0700 to 1800 hrs. The duration of submergence was 48 or 96 h. Each leaf was scored visually on a 0 to 10 scale with 0 being completely healthy and 10 indicating complete senescence. Three independent biological replicates of the submergence experiments were conducted during May and August. Samples were collected from three biological replicates for each genotype for scoring and RNA extraction. For QTL mapping, a single RIL family (Mo18w x B73) consisting of 180 lines from the NAM population was selected for phenotypic screening. Four independent biological replicates of the submergence experiments described above were conducted in the months of November, December, May, and June. Pots were submerged for 48 h and arranged in an alpha lattice design with two adjacent tubs comprising one replication. Phenotypic screening of RILs was performed at 48 h rather than 96 h due to the high sensitivity to submergence observed in many lines. A large portion of the RIL population experienced complete senescence when seedlings were submerged for longer than 48 h. Leaf senescence was scored on the first three leaves at 24 h after submergence.

### Visual scoring for leaf H_2_O_2_ and Superoxide

Plants at the V2 stage were submerged for a period of 96 h. To detect superoxide production, leaves were excised immediately after removing from submergence and placed in a 0.5 mg/mL nitroblue tetrazolium (NBT; Sigma-Aldrich, St. Louis, MO, USA) solution in 10mM potassium phosphate buffer (pH 7.6) and were incubated at 25ºC for 3h in complete darkness. For H_2_O_2_ visualization, leaf tissue was collected as described above, immersed in 1 mg/mL 3,3-diaminobenzidine tetrahydrochloride (DAB; Sigma-Aldrich, St. Louis, MO, USA) in 50 mM tris-acetate buffer (pH 5.0), and were incubated at 25ºC for 24 h in complete darkness. For each assay, the leaves were boiled in a 95% ethanol solution until all chlorophyll was removed, and were rehydrated in a 40% glycerol solution for 16 h at 25ºC.

### RNA Isolation

For submergence experiments, plants were removed from submergence tubs and the shoot tissue was cut approximately 1 cm above the soil line and snap frozen in liquid nitrogen. This process was completed within 10 seconds. RNA was extracted using the TRIzol method and quantified using Nanodrop8000 spectrophotometer (ThermoScientific, Wilmington, DE, USA). RNA cleanup was performed according to RNeasy MinElute Cleanup kit protocol (Qiagen, Valencia, CA, USA). RNA was eluted with 15 μl RNase-free water after on-column DNase1 treatment using the protocol described by the manufacturer.

### RNA sequencing

RNA sequencing was done using Illumina HiSeq 2000 sequencer. Prior to library construction, RNA integrity was analyzed using Agilent 2100 Bioanalyzer (Agilent Technologies, Palo Alto, CA, USA). RNAseq libraries were prepared using PolyA+ mRNA isolated from each RNA sample following standard Illumina RNASeq protocol. cDNA synthesis and library construction was done according to the manufactures guidelines. The 36 samples were distributed evenly across six lanes of the Illumina HiSeq2000 flow cell. On average, approximately 34,308,889 101 bp single-end reads were produced in each lane. The total number of reads obtained is provided as S5 Table. The raw data are publicly available through the National Center for Biotechnology Information Gene Expression Omnibus (GSE63429).

### RNA-seq differential expression analysis

Short reads were mapped against the B73 genome (ZmB73_RefGen_v2 from maizesequence.org) using Bowtie, allowing up to two base mismatches per read [61]. Reads mapped to multiple locations were discarded. Detailed mapping information for each sample is provided in S6 Table. Numbers of reads in genes were counted by the HTSeq-count tool using B73 gene annotations (ZmB73_5b_FGS; http://ftp.maizesequence.org/current/filtered-set/ZmB73_5b_FGS.gff.gz) and the “union” resolution mode was used [62]. For pair-wise comparisons, the edgeR package with TMM normalization method was used to analyze the numbers of reads aligned to genes and to identify differential expressed genes [63, 64]. A threshold value for fold-change of differential expression was set at log_2_ (fold-change) > 1 (two-fold actual value) and adjusted *p*-values < 0.001 for the null hypothesis.

### Identification of unannotated transcripts

Short reads were mapped against the B73 genome (ZmB73_RefGen_v2) using TopHat, allowing up to two base mismatches per read [65, 66]. Reads mapped to multiple locations were discarded. All mapped reads were analyzed with Cufflinks package to discover novel transcripts [66]. First, mapped reads for one replicate were assembled by Cufflinks to find expressed transcripts, and results of all replicates for the same genotype were merged together using the “cuffmerge” program. Second, these discovered transcripts were compared with B73 gene annotation (ZmB73_5a_WGS) with the “cuffcompare” program to find unannotated transcripts, which were considered as novel transcripts. Finally, the sequences of novel transcripts were extracted and blasted against the non-redundant (NR) library to determine if they are coding or non-coding genes [67]. Transcripts were considered to be potential protein coding genes if the e-value was less than 1 x 10^–8^. Differential expression analysis for unannotated transcripts was performed using edgeR as described above. The nucleotide sequence for *PFP1* (TCONS_00005048) showed 99.8% identity to that described by Guo et al [68] (JQ522972; S11 Fig).

### Transcription Factor and Gene Ontology Enrichment Analysis

To test whether transcription factor families were significantly enriched among differentially expressed genes the hypergeometric test was performed for each transcription factor family. Gene annotations were obtained from the Plant Transcription Factor DatabaseV3.0 [69]. The total number of genes annotated for each transcription factor family is listed in S1 Table and S2 Table.

Gene ontology enrichment analysis was conducted on sets of genes that were up- and down-regulated in response to submergence with respect to control plants. GO annotations for the complete proteome of B73 (Release 4a.53) were retrieved from AgriGO (Zea mays V5a; http://bioinfo.cau.edu.cn/agriGO/). Enrichment analysis was carried out using the Bioconductor package TopGO in R [70]. Only the “biological function” category was used for analysis. Enrichment tests were performed using the “classic” algorithm with Fisher’s exact test. Results of the GO enrichment analysis are provided as S4 File.

### Phylogenetic Analysis of ERF transcription factors

To determine phylogenetic relationships between maize, rice and Arabidopsis ERF genes multiple sequence alignment of the AP2 domain was performed using ClustalOMEGA (V1.2.1) with the following settings:—iterations 3—max-guidetree-iterations 3—max-hmm-iterations 3 [71] The AP2 domain(s) sequence was obtained from Plant Transcription Factor DatabaseV3.0 [69]. In some cases where multiple isoforms were reported for a single gene, only the longest isoform was selected. Sequences with more than one AP2 domain were excluded from the analysis. In total 238 maize proteins, 111 Arabidopsis and 104 rice proteins were used for alignment. Construction of the phylogenetic tree was performed using FastTree2 with Jones-Taylor-Thorton model of amino acid evolution and the “CAT” approximation was used to account for variation in rates across sites [72]. Maize genes were classified into ERF subfamilies based on similarity to Arabidopsis and rice ERF family members as reported by Nakano et al. [73]. A complete phylogenetic tree is provided as S5 File.

### Microarray Analysis

Microarray analysis for rice (*Oryza sativa*) and Arabidopsis (*Arabidopsis thaliana*) data was carried out as described by Placido et al. [74]. Briefly, the Bioconductor “affy” and “limma” packages were used for reading CEL files and normalizing microarray data [75]. The raw intensity values for the GeneChip arrays were background corrected, log_2_ transformed, and quantile normalized with robust multiarray average within the R package affy [76]. The statistical computation and assessment of differential expression were done with the empirical Bayes function, which moderates the SE values of estimated log fold changes toward a pooled SD value. P values were adjusted to the Benjamini and Hochberg method (BH) to control the false discovery rate. The top table function was used to list the genes according to the specified *p-value* of 0.05, log_2_ fold change of 1, and *p-value* adjust = BH.

### cDNA synthesis and quantitative PCR

First strand cDNA synthesis was performed using SuperScript VILO for real-time quantitative PCR (qPCR) (Invitrogen Corp., Carlsbad, CA, USA). First-strand cDNA was generated for the two-step quantitative real-time PCR (qRT-PCR) by following the protocol from Superscript VILO cDNA synthesis kit. An amount of 2 μg of RNA was used in the 20 μL reaction mixture. For the qPCR reaction, 3 μL of the diluted cDNA (1:20) was used in the 15 μL reaction mixture. In the qPCR reaction volume, 7.5 μL of LightCycler 480 SYBR Green I Mastermix was used (Roche Diagnostics, Indianapolis, IN, USA). The qRT-PCR was carried out using Roche LightCycle 480 II with the following parameter settings (Roche Diagnostics, Indianapolis, IN, USA): 95°C pre-incubation for 5 min, amplification was done for 40 cycles at 95°C for 20 sec and 60°C for 15 sec and extension at 72°C for 15 sec; the melting curve was set-up for 95°C, 65°C, 97°C; cooling was set-up at 40°C for 30 sec. We used two independent tissue samples, each sample having two technical replicates. Primer sequences are provided in S6 Table. Ubiquitin-conjugating enzyme (GRMZM2G102471) was used as an internal control and showed stable expression patterns in all samples. Relative expression was determined using the delta-delta Ct method [77].

### QTL Analysis

We conducted QTL analysis on mean leaf senescence score for three leaves from a single plant following submergence. On hundred sixty-six RILs common across 4 independent biological replicates were included in the analysis. The genetic map consisted of 1478 SNPs scored using genotype by sequencing (GBS). Data is from the Panzea database labeled AllZeaGBSv2.3 (http://www.panzea.org/lit/data_sets.html#genos). Missing data was previously phased and imputed using the algorithm of Swarts et al. [46]. Interval mapping was implemented with the R/qtl package and percent phenotypic variation was determined using the “fitqtl” function [78, 79]. The 5% significance level corresponded to a logarithm of odds threshold of 3.2 and were determined using 10,000 permutations of the phenotypic data. QTL intervals were further examined for significance by determining a 95% Bayes credible interval. Potential candidate genes within this credible interval underlying the major QTL on chromosome 6 were identified.

## Supporting Information

S1 Fig Graphical summary of visual scoring system used for phenotypic evaluation of submergence and dark-induced senescence. Leaves were scored visually on a 0–10 scale, with 0 indicating no visual stress symptoms and 10 indicating complete senescence.
(PDF)

S2 Fig Photograph describing leaf designations used for visual scoring. (PDF)

S3 Fig Photographs showing tolerant (top and bottom left) and sensitive (top and bottom right pictures) inbreds. Photographs were taken on day 5 of recovery after 96 h of submergence.
(PDF)

S4 Fig Venn diagrams showing differentially expressed genes at 24h and 72h after submergence. All genes showed significant differences in expression between submerged and control plants (p <0.001). Upregulation indicates higher expression in submerged samples.
(PDF)

S5 Fig Gene Ontology (GO) enrichment analysis of differentially expressed transcripts. (A, C). A subset of GO terms that were significantly enriched among genes showing similar expression patterns in tolerant or sensitive inbreds. Genes that are down-regulated in response to submergence are shown in panel A, while those that were up-regulated are shown in panel C. (B, D) A subset of GO terms that were significantly enriched among genes showing similar expression patterns in all inbreds. GO categories that are enriched among transcripts that are down-regulated in response to submergence are displayed in panel B, while those that are up-regulated in response to submergence are shown in panel D. All genes including in GO enrichment analysis showed significant differences in expression (FDR < 0.001). Scale indicates Log10 p-values determined using Fisher’s Exact test. Full results are provided as S4 File.
(PDF)

S6 Fig Expression of oxidative stress-related transcripts in comparisons between genotypes. (A). A subset of genes that are associated with oxidative stress responses and showed significant differences in expression between genotypes (FDR < 0.001). (B) Transcription factor genes belonging to families showing significant enrichment among significantly differentially expressed genes. These transcription factors were selected as they have been reported to be involved in leaf senescence in Arabidopsis. All genes including in GO enrichment analysis showed significant differences in expression (FDR < 0.001). Scale indicates Log2 fold change.
(PDF)

S7 Fig Expression of a subset of ERF transcription factors. (A) ERFs displaying significant differences in response to submergence. (B) ERFs displaying significant differences between inbreds. All genes showed significant differences in expression between submerged and control plants (FDR < 0.001). The scale indicates log2 fold-change. Up-regulation indicates higher expression in submerged samples.
(PDF)

S8 Fig An unrooted phylogenetic tree of maize, rice and Arabidopsis group B-2 (VII) ERF proteins. The amino acid sequence of AP2 domains were obtained from PlantTFDB [69] and aligned using ClustalOMEGA [70] and the phylogenetic tree was generated using the Jones-Taylor-Thorton model of amino acid evolution implemented in FastTree2 [71]. Maize genes were classified into ERF subfamilies based on similarity to Arabidopsis and rice ERF family members as reported by Nakano et al. [72]. Common names are provided for B-2 ERF proteins in Arabidopsis and rice. The protein sequences for rice were obtained from MSU Rice Genome Annotation Project Release 6.
(PDF)

S9 Fig CBF (C-REPEAT/DRE BINDING FACTOR) co-expression clusters. Each gene in the network is represented by a gray circle. The top cluster was seeded with GRMZM2G069146, while the cluster on the bottom was seeded with GRMZM2G069126.
(PDF)

S10 Fig Quantitative real-time PCR of a subset of *Subtol6* candidates. (a) Expression of *RELATED TO ABA-INSENSITIVE3(ABI3)/VIVIPAROUS1* in B73 and Mo18W. (b) Expression of *HEMOGLOBIN2 (HB2*; GRMZM2G168898) in B73 and Mo18W. Statistical significance was determined using a Student’s T-Test. Asterisks represent statistically significant differences between genotypes within each treatment: *** p < 0.001, ** p < 0.01.
(PDF)

S11 Fig Alignment of *PFP1* (TCONS_00005048) and JQ522972.1. (PDF)

S1 Table Transcription factor enrichment analysis from differentially expressed genes of each genotype at 24 h and 72 h after submergence. Comparisons were made between submerged and control samples.
(PDF)

S2 Table Transcription factor enrichment analysis for comparisons between genotypes at 24 h and 72 h after submergence. (PDF)

S3 Table Summary of comparative analysis of maize, Arabidopsis and rice submergence transcriptome analysis. (PDF)

S4 Table Maize varieties and subfamily classification [29]. NA: not included in the classification by McMullen et al., [29].
(PDF)

S5 Table Detailed mapping information for all 36 RNA-seq samples. Mapping was done using Bowtie and Tophat as described in Materials and Methods.
(PDF)

S6 Table List of Primers (PDF)

S1 File Genes displaying significant differences between treatments and genotypes (FDR < 0.001). (XLSX)

S2 File Full list of unannotated genes. (XLSX)

S3 File Genes within *Subtol6* displaying significant differences (FDR < 0.001) in transcript abundance between Mo18W and B73 under control, or after 24 and 72 h of submergence. (XLSX)

S4 File Gene ontology (GO) enrichment analysis of differentially expressed genes. (XLSX)

S5 File Phylogenetic tree of ERF transcription factors in rice, maize and Arabidopsis. (XLSX)
