# Supplementary material for: Genetic and Molecular Characterization of Submergence Response Identifies Subtol6 as a Major Submergence Tolerance Locus in Maize
Source: PLoS One. 2015 Mar 25;10(3):e0120385. doi: 10.1371/journal.pone.0120385 (PMC4373911; doi:10.1371/journal.pone.0120385)
Supplement: S1 Fig — Leaves were scored visually on a 0–10 scale, with 0 indicating no visual stress symptoms and 10 indicating complete senescence. (PDF) [file pone.0120385.s001.pdf]

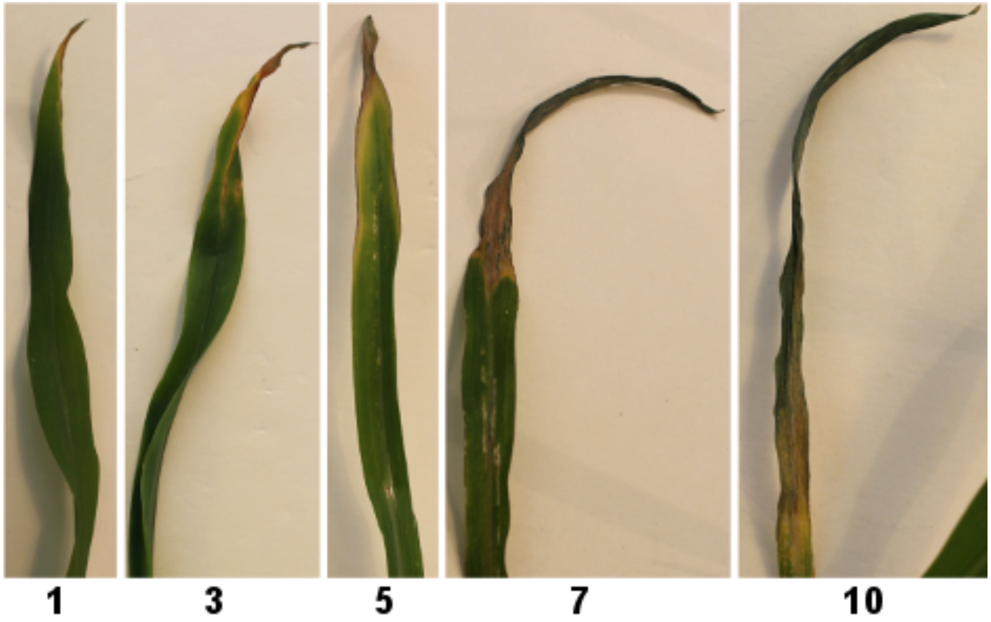

S1 Figure. Graphical summary of visual scoring system used for phenotypic evaluation of submergence and dark-induced senescence. Leaves were scored visually on a 0-10 scale, with 0 indicating no visual stress symptoms and 10 indicating complete senescence.
