# Supplementary figures and images for: Genetic and Molecular Characterization of Submergence Response Identifies Subtol6 as a Major Submergence Tolerance Locus in Maize
Source: PLoS One. 2015 Mar 25;10(3):e0120385. doi: 10.1371/journal.pone.0120385 (PMC4373911; doi:10.1371/journal.pone.0120385)

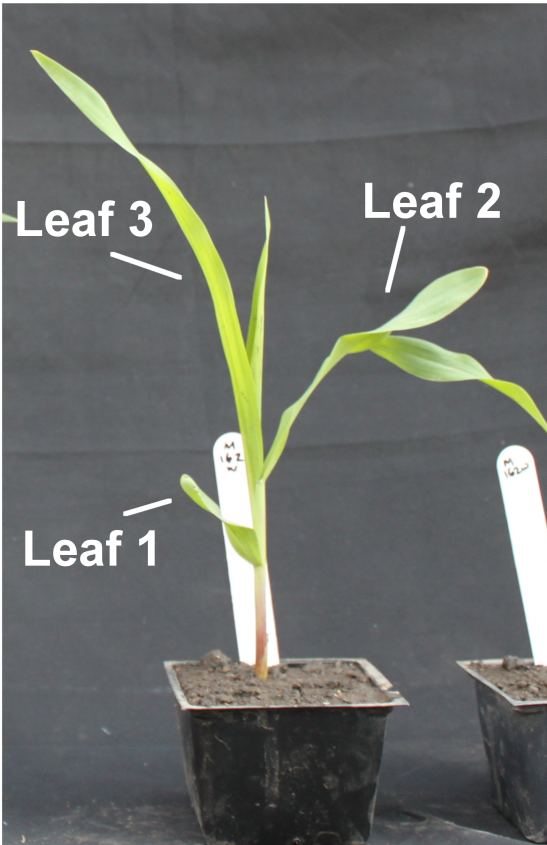

**S2 Figure.** Photograph describing leaf designations used for visual scoring.

Supplement: S2 Fig — (PDF) [file pone.0120385.s002.pdf]
