# Supplementary material for: Genetic and Molecular Characterization of Submergence Response Identifies Subtol6 as a Major Submergence Tolerance Locus in Maize
Source: PLoS One. 2015 Mar 25;10(3):e0120385. doi: 10.1371/journal.pone.0120385 (PMC4373911; doi:10.1371/journal.pone.0120385)
Supplement: S3 Fig — Photographs were taken on day 5 of recovery after 96 h of submergence. (PDF) [file pone.0120385.s003.pdf]

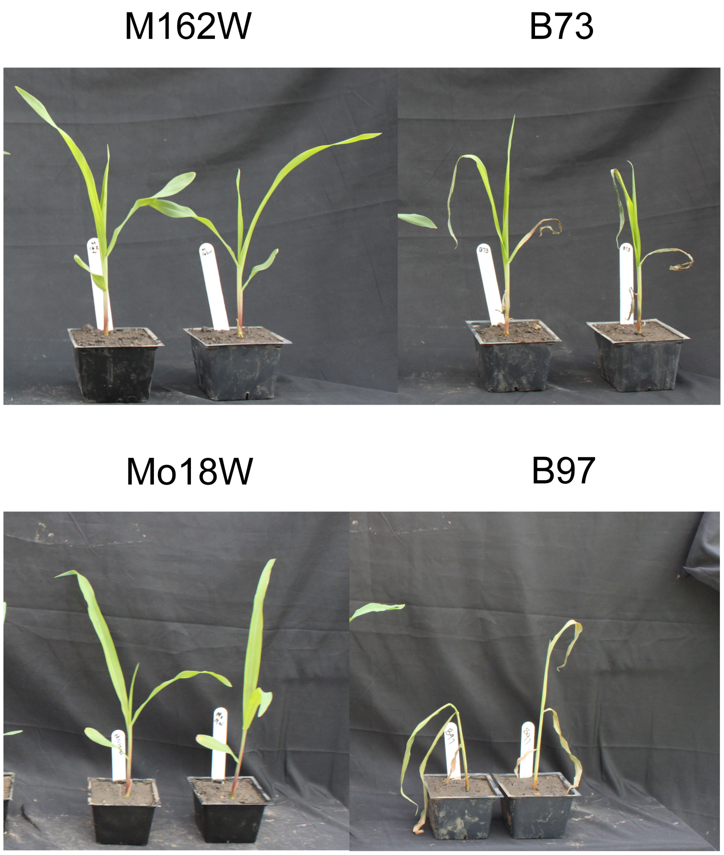

S3 Figure. Photographs showing tolerant (top and bottom left) and sensitive (top and bottom right pictures) inbreds. Photographs were taken on day 5 of recovery after 96 h of submergence.
