# Supplementary material for: Genetic and Molecular Characterization of Submergence Response Identifies Subtol6 as a Major Submergence Tolerance Locus in Maize
Source: PLoS One. 2015 Mar 25;10(3):e0120385. doi: 10.1371/journal.pone.0120385 (PMC4373911; doi:10.1371/journal.pone.0120385)
Supplement: S4 Fig — All genes showed significant differences in expression between submerged and control plants (p <0.001). Upregulation indicates higher expression in submerged samples. (PDF) [file pone.0120385.s004.pdf]

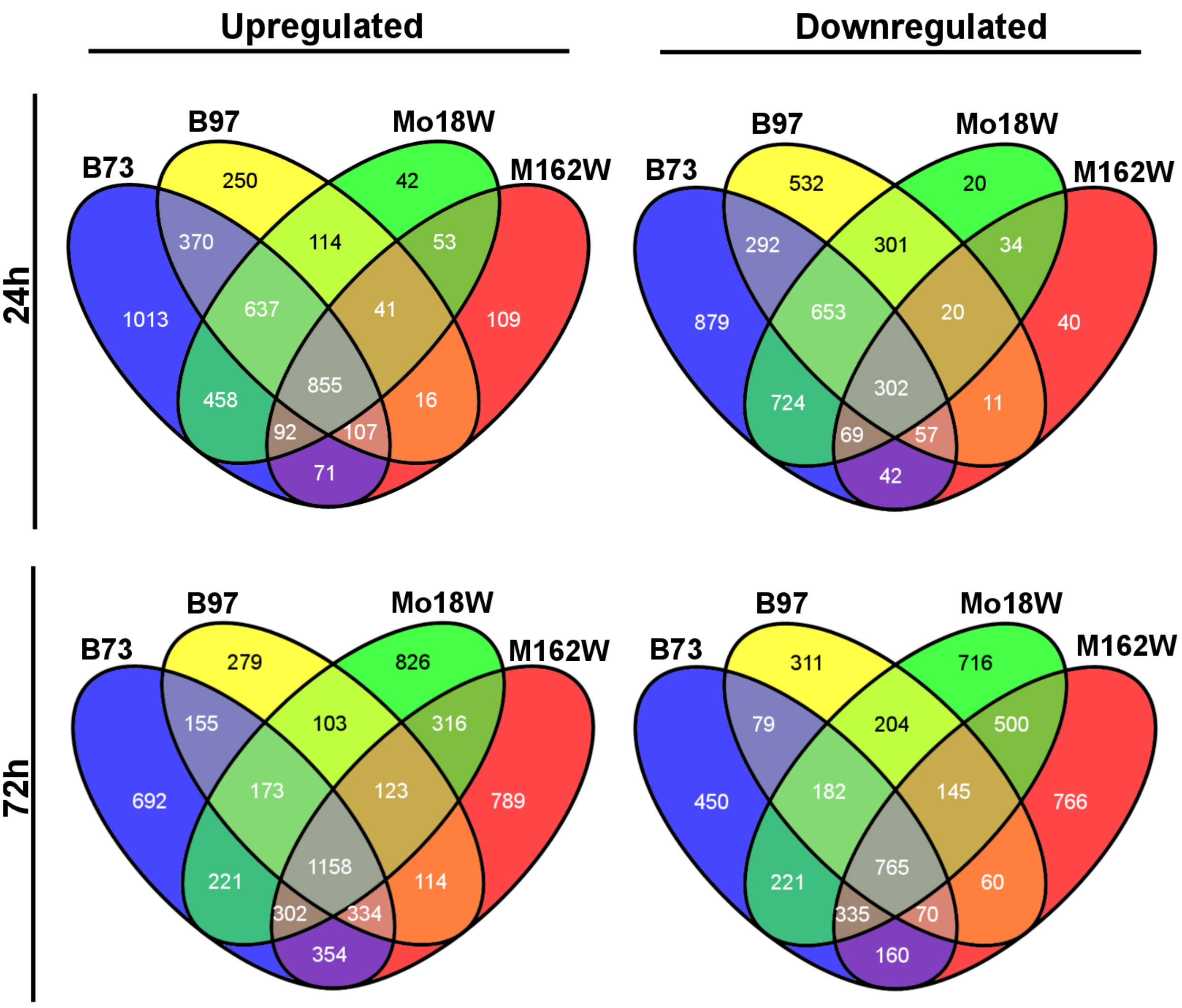

S4 Figure. Venn diagrams showing differentially expressed genes at 24h and 72h after submergence. All genes showed significant differences in expression between submerged and control plants ($p < 0.001$). Upregulation indicates higher expression in submerged samples
