# Supplementary material for: Genetic and Molecular Characterization of Submergence Response Identifies Subtol6 as a Major Submergence Tolerance Locus in Maize
Source: PLoS One. 2015 Mar 25;10(3):e0120385. doi: 10.1371/journal.pone.0120385 (PMC4373911; doi:10.1371/journal.pone.0120385)
Supplement: S5 Fig — (A, C). A subset of GO terms that were significantly enriched among genes showing similar expression patterns in tolerant or sensitive inbreds. Genes that are down-regulated in response to submergence are shown in panel A, while those that were up-regulated are shown in panel C. (B, D) A subset of GO terms that were significantly enriched among genes showing similar expression patterns in all inbreds. GO categories that are enriched among transcripts that are down-regulated in response to submergence are displayed in panel B, while those that are up-regulated in response to submergence are shown in panel D. All genes including in GO enrichment analysis showed significant differences in expression (FDR < 0.001). Scale indicates Log10 p-values determined using Fisher’s Exact test. Full results are provided as S4 File. (PDF) [file pone.0120385.s005.pdf]

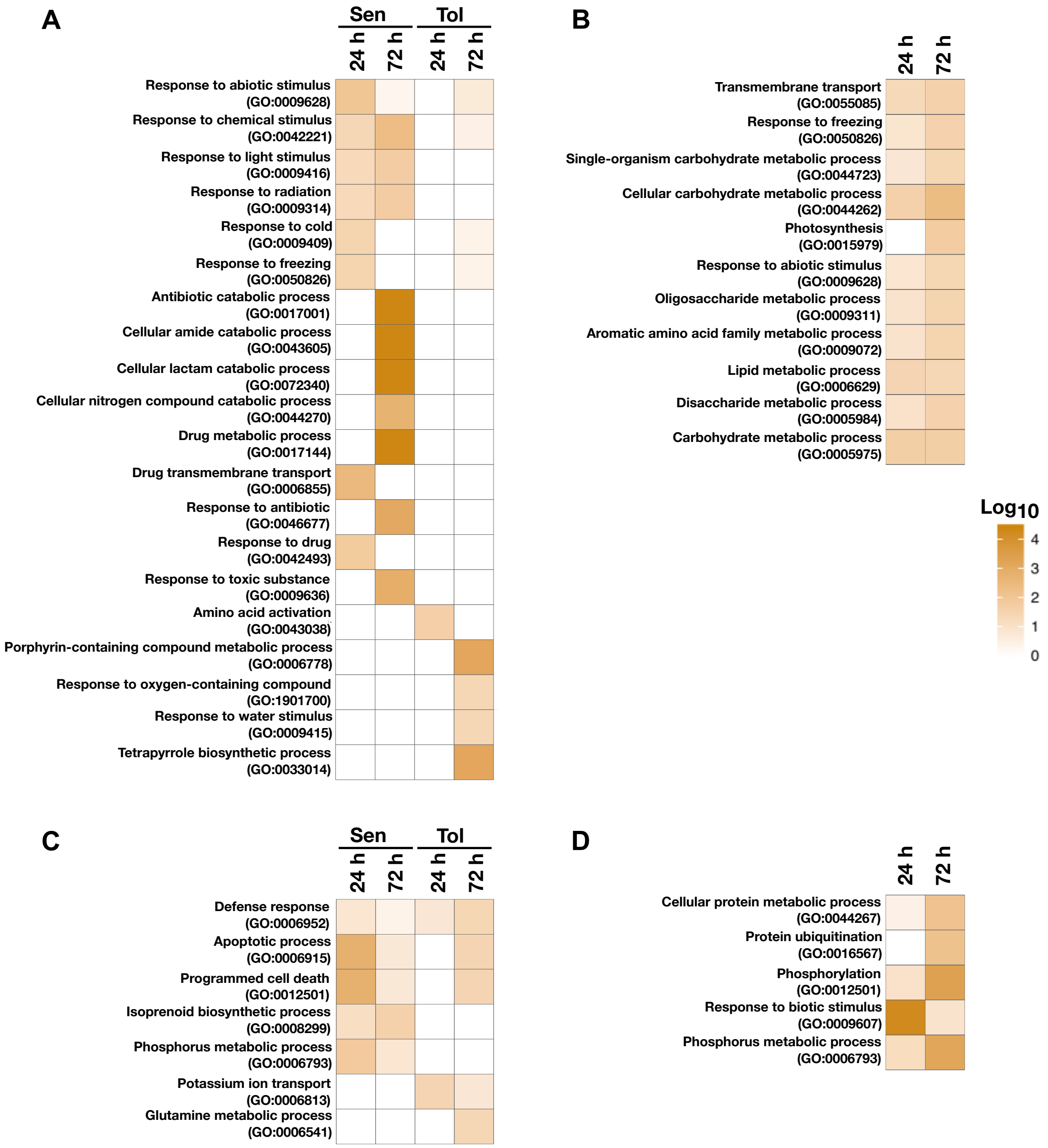

S5 Figure. Gene Ontology (GO) enrichment analysis of differentially expressed transcripts. (A, C). A subset of GO terms that were significantly enriched among genes showing similar expression patterns in tolerant or sensitive inbreds. Genes that are down-regulated in response to submergence are shown in panel A, while those that were up-regulated are shown in panel C. (B, D) A subset of GO terms that were significantly enriched among genes showing similar expression patterns in all inbreds. GO categories that are enriched among transcripts that are down-regulated in response to submergence are displayed in panel B, while those that are up-regulated in response to submergence are shown in panel D. All genes including in GO enrichment analysis showed significant differences in expression (FDR < 0.001). Scale indicates Log_{10} *p*-values determined using Fisher's Exact test. Full results are provided as Supplemental File S4.
