# Supplementary material for: Genetic and Molecular Characterization of Submergence Response Identifies Subtol6 as a Major Submergence Tolerance Locus in Maize
Source: PLoS One. 2015 Mar 25;10(3):e0120385. doi: 10.1371/journal.pone.0120385 (PMC4373911; doi:10.1371/journal.pone.0120385)
Supplement: S6 Fig — (A). A subset of genes that are associated with oxidative stress responses and showed significant differences in expression between genotypes (FDR < 0.001). (B) Transcription factor genes belonging to families showing significant enrichment among significantly differentially expressed genes. These transcription factors were selected as they have been reported to be involved in leaf senescence in Arabidopsis. All genes including in GO enrichment analysis showed significant differences in expression (FDR < 0.001). Scale indicates Log2 fold change. (PDF) [file pone.0120385.s006.pdf]

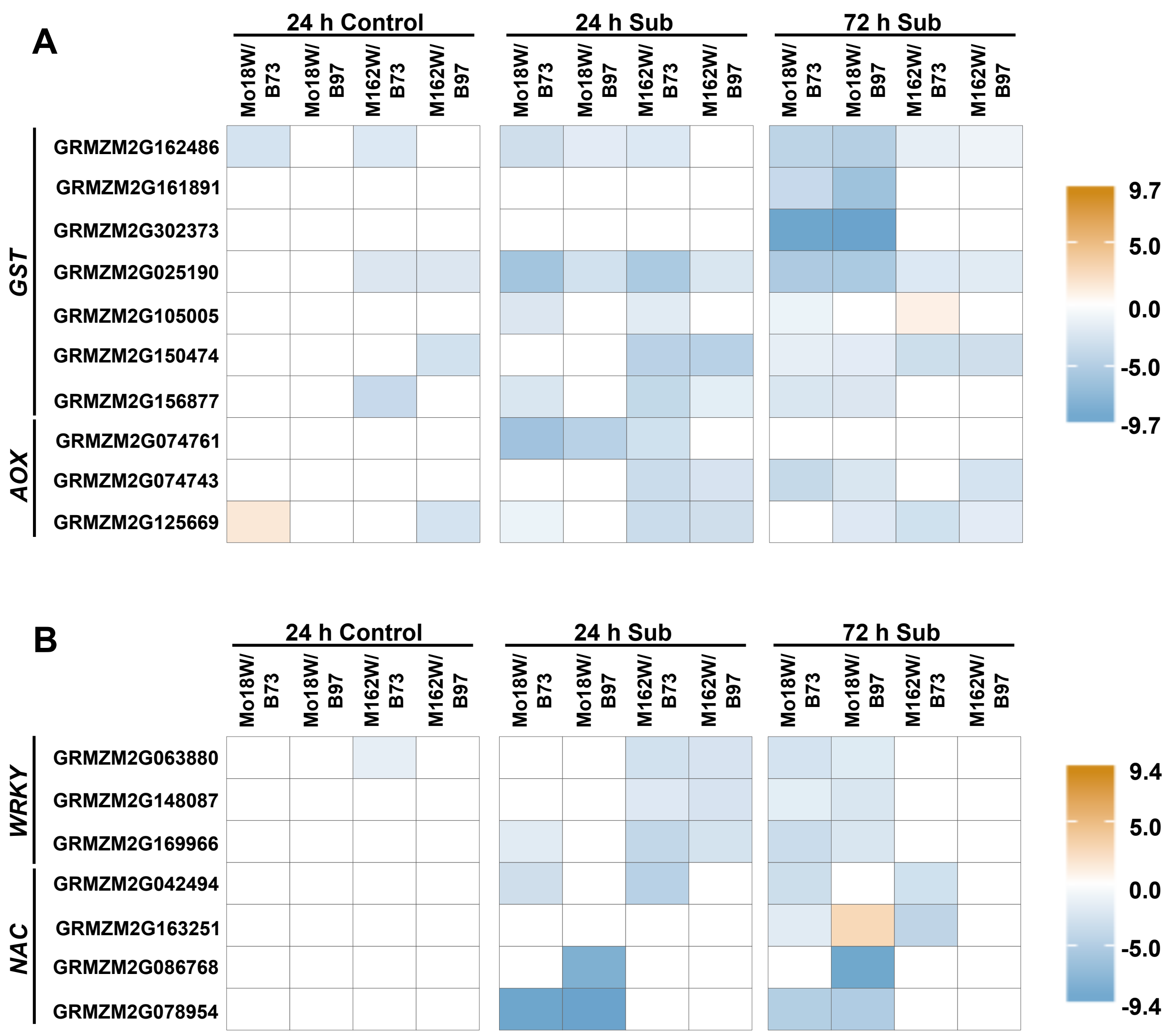

S6 Figure. Expression of oxidative stress-related transcripts in comparisons between genotypes. (A). A subset of genes that are associated with oxidative stress responses and showed significant differences in expression between genotypes (FDR < 0.001). (B) Transcription factor genes belonging to families showing significant enrichment among significantly differentially expressed genes. These transcription factors were selected as they have been reported to be involved in leaf senescence in Arabidopsis. All genes including in GO enrichment analysis showed significant differences in expression (FDR < 0.001). Scale indicates Log_2 fold change.
