# Supplementary material for: Genetic and Molecular Characterization of Submergence Response Identifies Subtol6 as a Major Submergence Tolerance Locus in Maize
Source: PLoS One. 2015 Mar 25;10(3):e0120385. doi: 10.1371/journal.pone.0120385 (PMC4373911; doi:10.1371/journal.pone.0120385)
Supplement: S7 Fig — (A) ERFs displaying significant differences in response to submergence. (B) ERFs displaying significant differences between inbreds. All genes showed significant differences in expression between submerged and control plants (FDR < 0.001). The scale indicates log2 fold-change. Up-regulation indicates higher expression in submerged samples. (PDF) [file pone.0120385.s007.pdf]

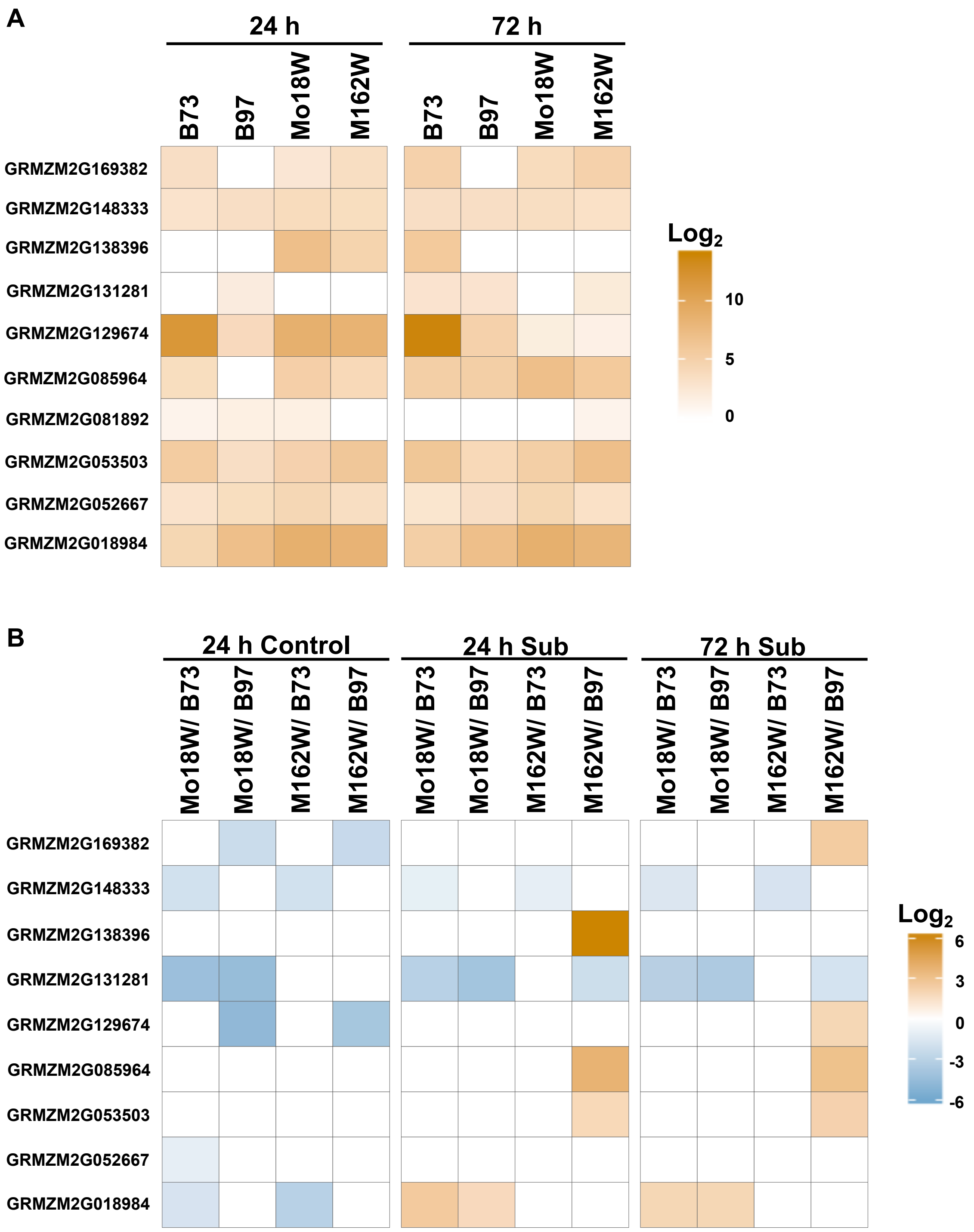

S7 Figure. Expression of a subset of ERF transcription factors. (A) ERFs displaying significant differences in response to submergence. (B) ERFs displaying significant differences between inbreds. All genes showed significant differences in expression between submerged and control plants (FDR <0.001). The scale indicates $\log_2$ fold-change. Up-regulation indicates higher expression in submerged samples.
