# Supplementary material for: Genetic and Molecular Characterization of Submergence Response Identifies Subtol6 as a Major Submergence Tolerance Locus in Maize
Source: PLoS One. 2015 Mar 25;10(3):e0120385. doi: 10.1371/journal.pone.0120385 (PMC4373911; doi:10.1371/journal.pone.0120385)
Supplement: S8 Fig — The amino acid sequence of AP2 domains were obtained from PlantTFDB [69] and aligned using ClustalOMEGA [70] and the phylogenetic tree was generated using the Jones-Taylor-Thorton model of amino acid evolution implemented in FastTree2 [71]. Maize genes were classified into ERF subfamilies based on similarity to Arabidopsis and rice ERF family members as reported by Nakano et al. [72]. Common names are provided for B-2 ERF proteins in Arabidopsis and rice. The protein sequences for rice were obtained from MSU Rice Genome Annotation Project Release 6. (PDF) [file pone.0120385.s008.pdf]

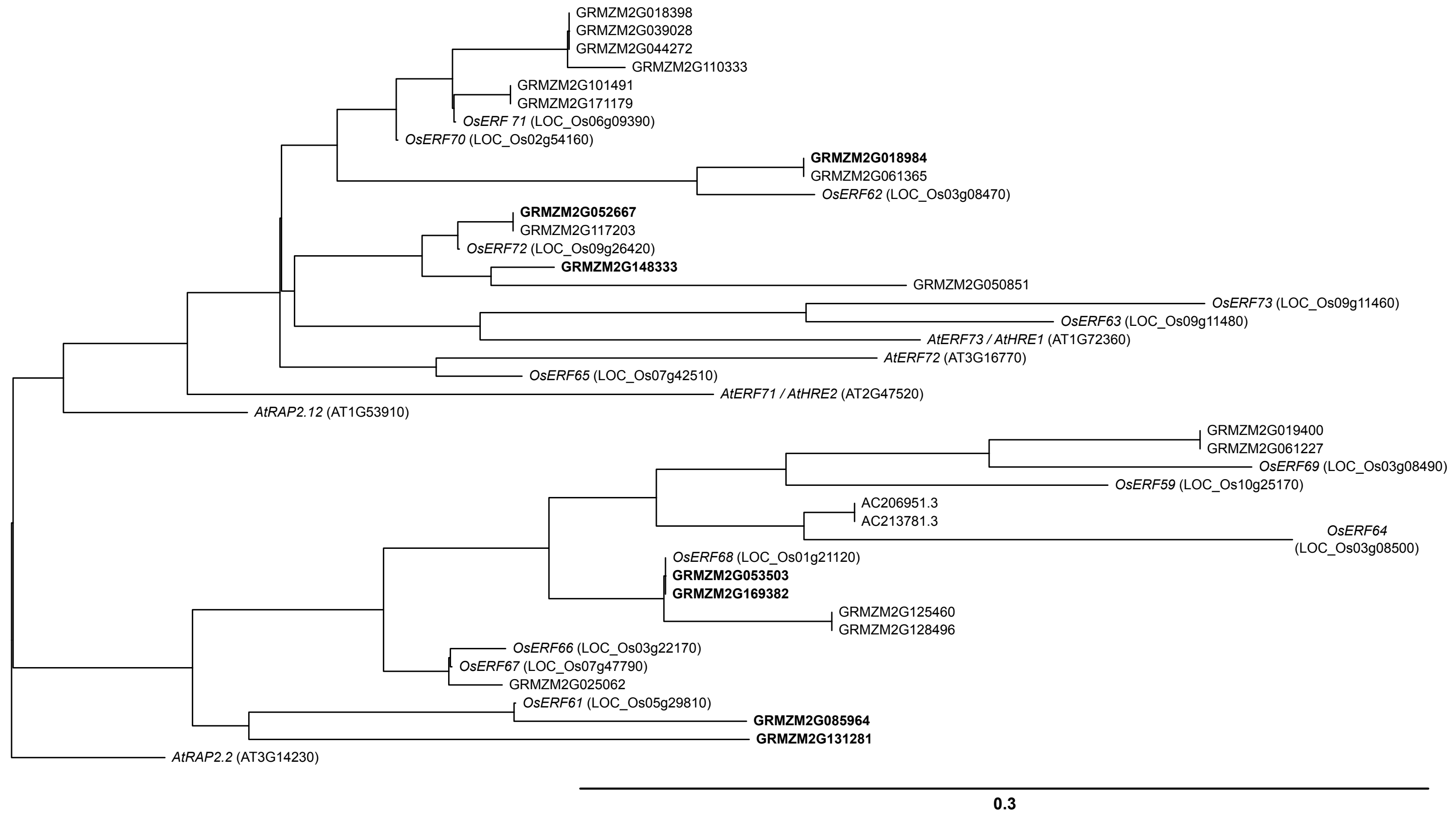

S8 Figure. An unrooted phylogenetic tree of maize, rice and Arabidopsis group B-2 (VII) ERF proteins. The amino acid sequence of AP2 domains were obtained from PlantTFDB (69) and aligned using ClustalOMEGA (70) and the phylogenetic tree was generated using the Jones-Taylor-Thorton model of amino acid evolution implemented in FastTree2 (71). Maize genes were classified into ERF subfamilies based on similarity to Arabidopsis and rice ERF family members as reported by Nakano et al. (72). Common names are provided for B-2 ERF proteins in Arabidopsis and rice. The protein sequences for rice were obtained from MSU Rice Genome Annotation Project Release 6.
