# Supplementary material for: Genetic and Molecular Characterization of Submergence Response Identifies Subtol6 as a Major Submergence Tolerance Locus in Maize
Source: PLoS One. 2015 Mar 25;10(3):e0120385. doi: 10.1371/journal.pone.0120385 (PMC4373911; doi:10.1371/journal.pone.0120385)
Supplement: S9 Fig — Each gene in the network is represented by a gray circle. The top cluster was seeded with GRMZM2G069146, while the cluster on the bottom was seeded with GRMZM2G069126. (PDF) [file pone.0120385.s009.pdf]

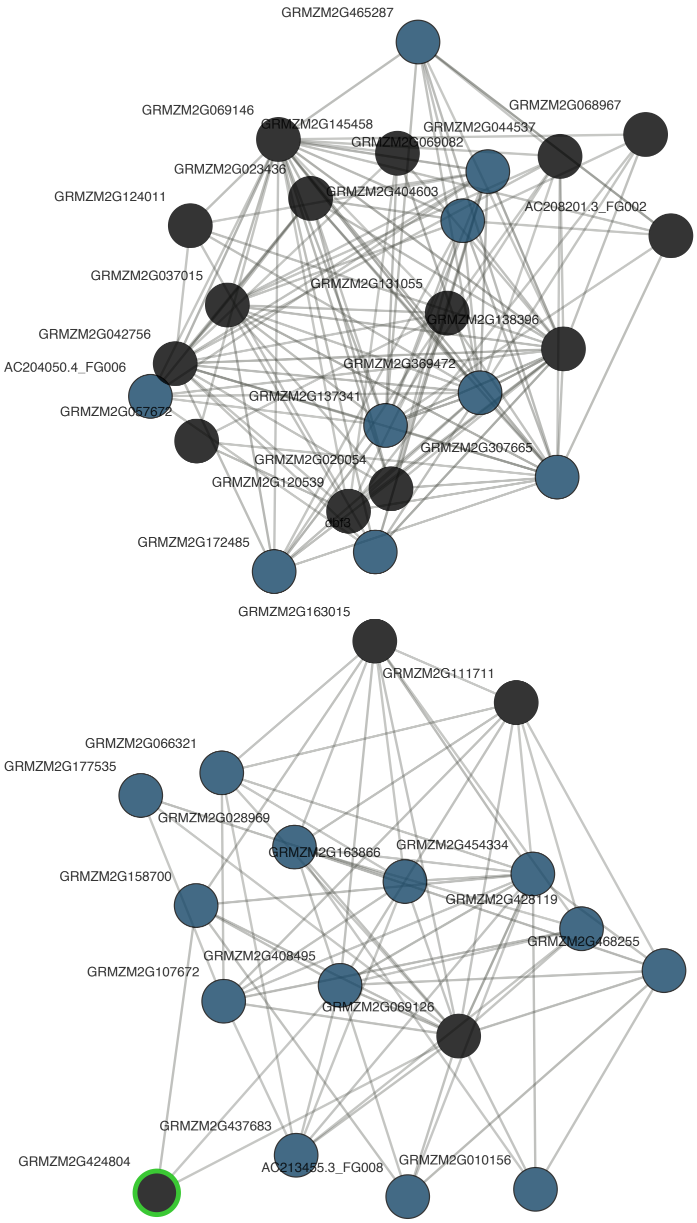

S9 Figure. CBF (*C-REPEAT/DRE BINDING FACTOR*) co-expression clusters. Each gene in the network is represented by a gray circle. The top cluster was seeded with GRMZM2G069146, while the cluster on the bottom was seeded with GRMZM2G069126.
