# Supplementary material for: Genetic and Molecular Characterization of Submergence Response Identifies Subtol6 as a Major Submergence Tolerance Locus in Maize
Source: PLoS One. 2015 Mar 25;10(3):e0120385. doi: 10.1371/journal.pone.0120385 (PMC4373911; doi:10.1371/journal.pone.0120385)
Supplement: S10 Fig — (a) Expression of RELATED TO ABA-INSENSITIVE3(ABI3)/VIVIPAROUS1 in B73 and Mo18W. (b) Expression of HEMOGLOBIN2 (HB2; GRMZM2G168898) in B73 and Mo18W. Statistical significance was determined using a Student’s T-Test. Asterisks represent statistically significant differences between genotypes within each treatment: *** p < 0.001, ** p < 0.01. (PDF) [file pone.0120385.s010.pdf]

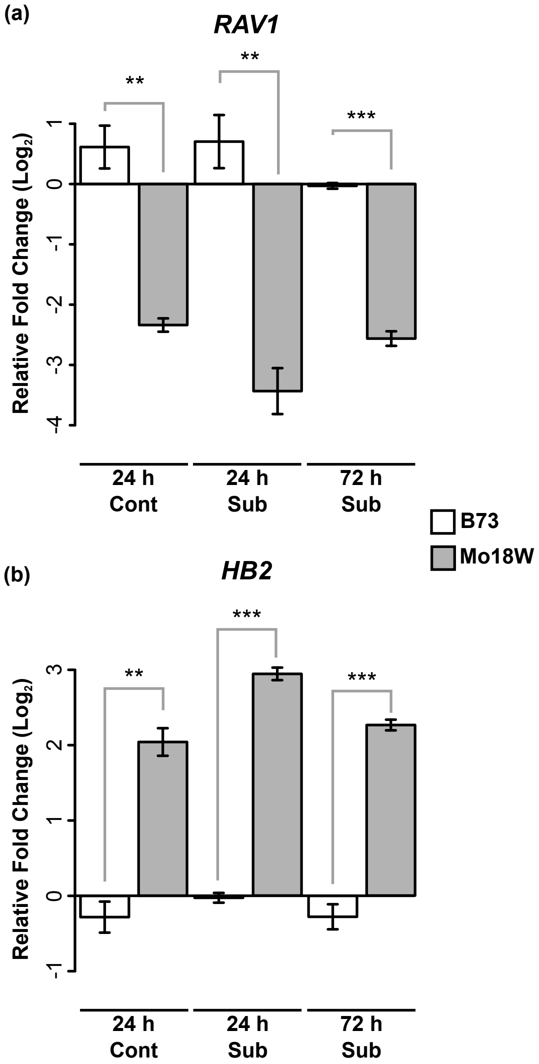

S10 Figure. Quantitative real-time PCR of a subset of *Subtol6* candidates. (a) Expression of *RELATED TO ABA-INSENSITIVE3(ABI3)/VIVIPAROUS1* in B73 and Mo18W. (b) Expression of *HEMOGLOBIN2* (*HB2*; GRMZM2G168898) in B73 and Mo18W. Statistical significance was determined using a Student's T-Test. Asterisks represent statistically significant differences between genotypes within each treatment: *** $p < 0.001$, ** $p < 0.01$.
