# Supplementary material for: Genetic and Molecular Characterization of Submergence Response Identifies Subtol6 as a Major Submergence Tolerance Locus in Maize
Source: PLoS One. 2015 Mar 25;10(3):e0120385. doi: 10.1371/journal.pone.0120385 (PMC4373911; doi:10.1371/journal.pone.0120385)
Supplement: S11 Fig — (PDF) [file pone.0120385.s011.pdf]

```
JQ522972.1      1330 AAATTTGATTGTGATTATGGCTACGTTCTTGGACATGTATGTTATCACAT   1379
                     |||||||||||.||||||||||||||||||||||||||||||||||||||
PFP                1 AAATTTGATTGCGATTATGGCTACGTTCTTGGACATGTATGTTATCACAT     50

JQ522972.1      1380 CGTAGCGGCTGGTTTGAACGGCTACATGGCCACTGTGACCAACCTTAAGA   1429
                     ||||||||||||||||||||||||||||||||||||||||||||||||||
PFP               51 CGTAGCGGCTGGTTTGAACGGCTACATGGCCACTGTGACCAACCTTAAGA    100

JQ522972.1      1430 GTCCAGTAAACAAATGGAAATGTGGTGCTGCTCCCATTACATCTATGATG   1479
                     ||||||||||||||||||||||||||||||||||||||||||||||||||
PFP              101 GTCCAGTAAACAAATGGAAATGTGGTGCTGCTCCCATTACATCTATGATG    150

JQ522972.1      1480 ACTGTGAAGAGATGGTCACGTGGTGCTGCCACCAGTCAAATCGGTAAGCC   1529
                     ||||||||||||||||||||||||||||||||||||||||||||||||||
PFP              151 ACTGTGAAGAGATGGTCACGTGGTGCTGCCACCAGTCAAATCGGTAAGCC    200

JQ522972.1      1530 TGCTGTCCACATGGCGAGCGTTGACTTAAAAGGAAAAGCATATGAGTTGT   1579
                     ||||||||||||||||||||||||||||||||||||||||||||||||||
PFP              201 TGCTGTCCACATGGCGAGCGTTGACTTAAAAGGAAAAGCATATGAGTTGT    250

JQ522972.1      1580 TGAGACAAAATTCTTCCATCTTCATGATGGAAGACATCTACCGAAACCCT   1629
                     ||||||||||||||||||||||||||||||||||||||||||||||||||
PFP              251 TGAGACAAAATTCTTCCATCTTCATGATGGAAGACATCTACCGAAACCCT    300

JQ522972.1      1630 GGACCACTTCAGTTCGAAGGGTCGGGTGCTGATACAAAACCTATTTCATT   1679
                     ||||||||||||||||||||||||||||||||||||||||||||||||||
PFP              301 GGACCACTTCAGTTCGAAGGGTCGGGTGCTGATACAAAACCTATTTCATT    350

JQ522972.1      1680 GTGTGTTGAAGATCAGGATTACATGGGAAGAATCAAGAAGTTGCAGGAGT   1729
                     ||||||||||||||||||||||||||||||||||||||||||||||||||
PFP              351 GTGTGTTGAAGATCAGGATTACATGGGAAGAATCAAGAAGTTGCAGGAGT    400

JQ522972.1      1730 ATTTGGAGAAGGTGAAAAGCATTGTGAAGCCCGGGTGCTCGCAGGATGTC   1779
                     ||||||||||||||||||||||||||||||||||||||||||||||||||
PFP              401 ATTTGGAGAAGGTGAAAAGCATTGTGAAGCCCGGGTGCTCGCAGGATGTC    450

JQ522972.1      1780 CTTAAAGCAGCGGTAAGCGCCATGGCTTCTGTGACGGAGATGTTGACCAT   1829
                     ||||||||||||||||||||||||||||||||||||||||||||||||||
PFP              451 CTTAAAGCAGCGGTAAGCGCCATGGCTTCTGTGACGGAGATGTTGACCAT    500

JQ522972.1      1830 CATGTCTTCCCTTTCATTTAGTGGACAGGCGACCATCTGA   1869
                     ||||||||||||||||||||||||||||||||||||||||
PFP              501 CATGTCTTCCCTTTCATTTAGTGGACAGGCGACCATCTGA    540
```

S11 Figure. Alignment of *PFP1* (TCONS_00005048) and JQ522972.1.
