# Supplementary material for: Genetic and Molecular Characterization of Submergence Response Identifies Subtol6 as a Major Submergence Tolerance Locus in Maize
Source: PLoS One. 2015 Mar 25;10(3):e0120385. doi: 10.1371/journal.pone.0120385 (PMC4373911; doi:10.1371/journal.pone.0120385)
Supplement: S1 Table — Comparisons were made between submerged and control samples. (PDF) [file pone.0120385.s012.pdf]

S1 Table. Transcription factor enrichment analysis from differentially expressed genes of each genotype at 24 h and 72 h after submergence. Comparisons were made between submerged and control samples.

	24h								72h								No. of Annotated TFs
	B73		B97		Mo18W		M162W		B73		B97		Mo18W		M162W		
	Down	Up	Down	Up	Down	Up	Down	Up	Down	Up	Down	Up	Down	Up	Down	Up	
ABI3VP1	0.711	1	0.601	1	0.559	1	1	1	0.596	1	0.458	1	0.298	1	0.238	1	18
Alfin-like	0.265	1	0.310	1	1	1	0.072	0.414	0.124	0.413	0.142	1	0.425	1	0.329	0.850	274
AP2-EREBP	0.177	1	1	1	0.319	1	1	1	1	1	1	1	1	1	0.907	1	48
ARF	0.838	0.982	1	0.742	0.887	0.945	1	0.827	1	0.984	1	0.947	1	1	1	0.985	8
ARID	1	0.965	1	1	1	1	1	1	0.867	0.856	1	0.914	1	0.814	0.880	1	12
ARR-B	0.995	0.998	1	1	1	1	1	1	1	0.990	1	1	0.950	0.998	0.983	0.999	48
AUX/IAA	0.505	0.965	0.871	1	1	0.911	0.369	1	0.045	0.657	0.744	0.695	1	1	0.053	0.857	6
BBR/BPC	0.314	1	1	1	1	1	1	1	1	1	1	1	1	1	1	1	9
BES1	0.314	1	1	1	1	1	1	1	1	1	1	1	1	1	1	1	222
bHLH	0.410	0.912	0.439	1	0.643	0.787	1	1	0.429	0.921	0.810	0.949	0.801	0.720	0.723	0.922	13
BSD	1	1	1	1	1	1	1	1	1	1	1	1	1	1	1	1	138
bZIP	4.98E-04	0.910	0.114	0.756	1.29E-04	0.812	0.039	0.788	9.57E-03	0.924	0.537	0.525	0.008	0.641	0.032	0.968	25
C2C2-CO-like	0.380	0.806	1	1	0.216	0.693	1	1	1	0.816	1	0.698	1	1	0.645	0.817	51
C2C2-Dof	1	0.178	1	1	1	1	1	1	1	0.183	1	1	1	0.167	1	0.184	46
C2C2-GATA	0.855	0.002	0.988	1.17E-04	0.818	0.014	0.013	0.295	0.587	0.001	0.929	0.007	0.643	0.234	0.785	2.29E-04	17
C2C2-YABBY	1	1	1	1	1	1	1	1	1	1	1	1	1	0.156	1	0.183	161
C2H2	1	1	1	1	1	1	1	1	1	1	1	1	1	1	1	1	102
C3H	0.739	0.329	0.155	0.742	0.665	0.576	0.872	0.286	0.349	0.596	0.063	0.461	0.737	0.441	0.685	0.491	7
CAMTA	1	0.693	1	1	1	1	1	1	1	0.330	1	1	0.584	1	1	1	62
CCAAT	0.784	0.449	0.630	0.679	1	0.719	1	0.789	1	0.975	0.416	0.725	0.734	0.838	0.894	0.698	4
Coactivator p15	1	1	1	1	1	1	1	1	1	1	1	1	1	1	1	1	18
CPP	0.600	1	0.781	1	1	1	1	1	0.777	1	0.637	1	0.844	1	1	1	6
CSD	1	1	1	1	1	1	1	1	1	1	1	1	1	0.548	1	1	9
DBP	0.447	1	0.066	1	0.054	1	1	1	0.064	1	0.254	1	0.414	1	0.070	1	4
DDT	0.140	0.476	0.015	0.665	0.589	0.321	1	0.512	0.069	0.816	0.486	0.698	8.65E-04	0.181	0.017	0.817	19
E2F-DP	1	1	1	1	1	1	1	1	1	1	1	1	1	1	1	1	13
EIL	0.016	1	1	1	0.017	1	1	1	1	1	1	1	1	0.830	1	1	29
FAR1	1	0.009	1	0.011	1	0.014	1	0.117	1	0.044	1	0.015	1	0.032	1	0.010	19
FHA	1	1	0.910	1	1	1	1	1	1	0.983	0.799	1	1	0.879	1	0.983	73
G2-like	1	0.573	1	1	1	0.458	1	1	1	1	1	0.463	1	0.548	1	1	13
GeBP	1	1	0.301	1	1	1	1	1	1	1	1	1	0.065	1	0.048	1	56
GNAT	0.371	1	0.111	0.931	0.220	0.997	1	0.992	0.623	1	0.028	0.997	0.092	0.991	0.132	1	95
GRAS	0.048	1	0.022	1	0.434	1	1	1	1	0.661	0.064	1	1	1	1	0.282	16
GRF	0.926	0.080	0.565	0.146	0.820	0.177	1	0.152	0.557	0.383	0.723	0.183	1	1	0.585	0.386	166
HB	0.802	0.860	1	0.368	0.656	0.405	1	1	1	0.869	1	0.411	0.420	0.840	0.119	0.870	16
HMG	0.002	0.792	0.001	0.727	0.007	0.534	0.246	0.174	2.73E-06	0.189	2.95E-05	0.185	4.95E-08	0.165	0.002	0.621	2
HRT	1	1	1	1	1	1	1	1	1	1	1	1	1	1	1	0.238	38
HSF	0.563	0.016	0.893	0.019	0.967	0.029	1	0.505	0.302	0.019	0.922	0.031	0.162	0.106	0.540	0.044	3
IWS1	1	1	1	1	1	1	1	1	1	1	1	1	1	1	1	1	25
Jumonji	0.977	0.068	0.938	0.170	1	0.393	1	0.585	0.935	0.161	1	0.402	1	0.110	1	0.476	2
LFY	1	1	1	1	1	1	1	1	1	1	1	1	1	1	1	1	11
LIM	0.146	0.197	1	0.433	1	0.118	1	1	0.087	0.585	1	0.463	1	0.177	0.095	0.586	69
LOB	1	1	1	1	1	1	1	1	1	1	1	1	1	1	1	1	6
LUG	1	1	1	1	1	1	1	1	1	1	1	1	1	1	1	1	97
MADS	1	0.693	1	1	1	1	1	1	1	0.704	1	1	1	0.288	1	0.706	4
MBF1	1	1	1	1	1	1	1	1	1	1	1	1	1	1	1	1	2
MED6	1	1	1	1	1	1	1	1	1	1	1	1	1	0.115	1	0.127	2
MED7	1	1	1	1	1	1	1	1	1	1	1	1	1	1	1	1	30
mTERF	0.159	0.873	0.062	0.688	0.123	0.728	0.391	0.795	0.355	0.884	0.174	0.929	9.42E-04	0.966	0.659	0.977	193
MYB	0.991	3.85E-05	0.988	9.41E-04	0.977	7.71E-04	1	0.075	0.987	2.17E-06	0.986	3.05E-05	0.477	1.11E-04	0.959	7.61E-06	128
MYB-related	1	0.140	0.894	0.215	0.865	0.738	0.397	0.801	1	0.155	0.777	0.263	1	1	0.903	0.721	158
NAC	0.302	0.748	0.197	1	0.526	1	1	1	0.562	1	1	1	0.640	1	0.581	0.760	55
OFP	0.740	0.514	0.169	0.374	0.717	0.301	0.123	0.550	0.787	0.689	0.884	0.629	0.964	0.113	0.488	0.115	146
Orphans	0.864	1.74E-06	0.963	2.61E-06	0.926	1.25E-05	0.715	2.07E-11	0.740	2.43E-05	0.736	1.16E-04	0.542	2.45E-04	0.430	5.68E-07	3
PBF-2-like	0.465	1	0.116	1	0.820	1	1	1	0.850	1	0.723	1	0.673	0.547	1	0.962	61
PHD	0.236	1	1	1	0.163	1	1	1	1	1	1	1	1	1	1	1	21
PLATZ	1	1	1	1	1	1	1	1	1	1	1	1	1	1	1	1	3
Pseudo ARR-B	1	0.230	1	0.160	1	0.172	1	0.108	1	0.237	1	0.174	1	1	1	0.238	7
RB	0.641	0.712	1	1	1	1	1	1	0.526	0.723	0.397	1	0.603	0.687	0.544	0.725	3
Rcd1-like	1	1	1	1	1	1	1	1	1	1	1	1	1	1	1	1	22
RWP-RK	0.792	0.470	0.569	0.641	0.479	0.328	1	0.553	0.356	0.502	0.504	0.338	0.207	0.077	0.229	0.668	2
S1Fa-like	1	1	1	1	1	1	1	1	1	1	1	1	1	1	1	1	38
SBP	0.194	1	1	1	0.132	1	1	1	1	1	1	1	1	1	1	1	57
SET	0.203	0.649	0.022	1	0.105	0.530	1	1	0.467	0.082	0.347	1	0.174	0.624	0.484	0.282	10
Sigma70-like	0.061	0.743	0.806	1	0.013	1	1	1	1	0.759	1	1	1	1	0.818	0.761	41
SNF2	0.135	0.009	0.184	0.487	0.042	0.007	0.044	0.290	0.056	0.233	0.263	0.259	0.268	0.184	0.459	0.010	1
SOH1	1	1	1	1	1	1	1	1	1	1	1	1	1	1	1	1	15
SRS	1	1	1	1	1	1	1	1	1	1	1	1	1	1	1	1	25
SWI/SNF-BAF60b	1	1	1	1	1	1	1	1	1	1	1	1	1	1	1	1	5
SWI/SNF-SWI3	0.974	0.442	0.441	0.536	0.979	0.321	1	0.840	0.953	0.483	0.803	0.771	0.785	0.353	0.478	0.619	8
TAZ	1	1	1	1	1	1	1	1	1	1	1	1	1	1	1	1	54
TCP	0.219	1	0.032	1	0.021	1	1	1	0.260	1	0.031	1	0.172	1	0.561	0.815	50
Tify	0.149	1	0.004	1	0.101	1	2.30E-04	1	0.111	1	1	1	1	1	0.005	1	51
TRAF	0.908	0.778	0.518	0.855	0.197	1	0.327	1	1	0.156	1	0.879	0.883	0.933	0.839	0.950	44
Trihelix	1	0.480	1	0.354	1	0.376	1	0.249	1	0.491	1	0.380	1	0.457	1	0.493	21
TUB	0.322	0.764	0.585	0.241	0.177	0.646	0.179	0.468	0.579	1	0.443	0.273	0.657	0.137	0.598	0.428	3
ULT	1	1	1	1	1	1	1	1	1	1	1	1	1	1	1	1	5
VOZ	1	1	1	1	1	1	1	1	1	1	1	1	1	1	1	1	160
WRKY	0.356	0.917	0.964	0.918	0.660	0.850	0.689	0.707	0.221	0.741	0.660	0.554	0.948	0.885	0.898	0.931	23
zf-HD	0.182	0.851	0.154	0.826	0.249	0.661	0.137	0.616	0.060	0.865	0.041	0.862	0.263	0.064	0.073	0.715	60
