# Supplementary material for: Genetic and Molecular Characterization of Submergence Response Identifies Subtol6 as a Major Submergence Tolerance Locus in Maize
Source: PLoS One. 2015 Mar 25;10(3):e0120385. doi: 10.1371/journal.pone.0120385 (PMC4373911; doi:10.1371/journal.pone.0120385)
Supplement: S2 Table — (PDF) [file pone.0120385.s013.pdf]

S2 Table. Transcription factor enrichment analysis for comparisons between genotypes at 24 h and 72 h after submergence.

	24h								72h								No. of Annotated TFs
	Mo18W vs B73		Mo18W vs B97		M162W vs B73		M162W vs B97		Mo18W vs B73		Mo18W vs B97		M162W vs B73		M162W vs B97		
	Down	Up	Down	Up	Down	Up	Down	Up	Down	Up	Down	Up	Down	Up	Down	Up	
**ABI3VP1**	0.786	0.572	0.819	0.810	1	0.009	1	0.395	0.649	0.669	0.706	0.819	1	0.021	0.909	0.104	18
**Alfin-like**	1	1	1	0.390	1	1	1	0.500	1	0.281	1	0.400	1	1	1	1	274
**AP2-EREBP**	0.149	0.012	0.354	0.465	0.140	0.522	0.161	0.334	0.071	0.744	0.681	0.891	0.358	0.310	0.034	0.420	48
**ARF**	1	0.493	0.745	1	1	0.486	1	1	0.935	0.011	0.585	0.389	0.330	1	1	0.062	8
**ARID**	1	1	1	1	1	1	1	1	1	1	1	1	1	1	1	1	12
**ARR-B**	1	1	1	1	1	1	1	1	1	0.019	1	0.288	1	0.278	1	0.205	48
**AUX/IAA**	1	1	1	1	1	1	1	1	1	1	1	1	1	1	1	1	6
**BBR/BPC**	1	1	1	0.152	1	1	1	0.206	1	1	1	0.156	1	0.150	1	0.109	9
**BES1**	1	1	1	0.023	0.290	1	1	0.292	1	1	1	0.225	0.201	0.217	0.300	1	222
**bHLH**	0.101	0.960	0.088	0.728	0.971	0.957	0.932	0.038	0.686	0.777	0.548	0.043	0.977	0.014	0.871	0.615	13
**BSD**	1	1	1	0.300	1	1	1	0.393	0.521	0.212	0.416	0.308	1	0.298	1	1	138
**bZIP**	0.330	0.124	0.542	0.317	0.412	0.038	0.877	0.005	0.780	0.239	0.488	0.185	0.249	0.163	0.627	0.045	25
**C2C2-CO-like**	0.334	0.047	0.508	0.029	0.239	0.292	0.479	0.242	0.155	0.368	0.268	0.153	0.022	1	0.070	1	51
**C2C2-Dof**	0.221	1	0.766	0.158	0.040	1	0.737	1	0.146	0.609	0.149	0.766	0.357	1	0.046	1	46
**C2C2-GATA**	0.172	0.478	0.730	0.355	1	0.471	1	0.093	0.038	0.571	0.561	0.369	0.683	0.350	0.840	1	17
**C2C2-YABBY**	0.563	1	1	0.373	0.477	1	1	1	0.618	1	1	1	1	1	0.490	1	161
**C2H2**	0.077	0.901	0.005	0.143	0.145	0.654	0.053	0.734	0.268	0.565	0.325	0.078	0.098	0.433	0.600	0.818	102
**C3H**	0.721	0.419	0.946	0.770	0.901	0.049	0.932	0.747	0.926	0.281	0.924	0.785	1	0.765	0.765	0.127	7
**CAMTA**	1	1	1	1	1	1	1	1	1	1	1	1	1	1	1	1	62
**CCAAT**	0.570	0.584	0.521	0.504	0.681	0.577	0.803	0.685	0.122	0.681	0.241	0.829	0.453	1	0.434	0.698	4
**Coactivator p15**	1	1	1	1	1	1	1	1	1	1	1	1	1	1	1	1	18
**CPP**	1	1	1	1	1	1	1	1	0.639	1	1	1	1	1	0.510	1	6
**CSD**	1	0.081	1	1	1	1	1	1	1	0.104	1	1	1	1	1	1	9
**DBP**	1	1	1	1	1	1	1	1	0.399	1	0.311	1	0.201	1	1	1	4
**DDT**	1	1	1	1	1	1	1	1	1	1	1	1	1	1	1	1	19
**E2F-DP**	1	1	1	1	1	1	1	1	1	1	1	1	1	1	1	1	13
**EIL**	0.469	0.013	0.308	0.300	0.391	0.164	0.287	0.393	0.521	0.212	0.416	0.308	0.276	1	0.402	1	29
**FAR1**	1	1	1	1	1	1	1	1	1	1	1	1	1	1	1	1	19
**FHA**	0.603	1	1	1	0.157	1	0.390	1	0.659	0.294	1	0.097	1	1	0.529	1	73
**G2-like**	0.056	0.645	0.145	0.134	0.053	0.263	0.288	0.523	0.104	0.740	0.572	0.610	0.540	0.310	0.022	0.756	13
**GeBP**	0.469	1	1	1	1	1	1	0.393	1	1	1	1	1	1	1	1	56
**GNAT**	1	1	1	1	1	1	1	1	1	1	1	1	1	1	1	1	95
**GRAS**	0.472	0.145	0.048	0.255	0.063	0.139	0.003	0.286	0.148	0.007	0.014	0.272	0.203	0.471	0.507	0.033	16
**GRF**	1	1	1	0.356	0.457	1	1	0.120	0.596	1	1	0.365	0.329	0.353	0.469	1	166
**HB**	0.049	0.199	0.851	0.160	0.877	0.671	0.933	0.093	0.985	0.586	0.993	0.496	0.386	0.284	0.894	1	16
**HMG**	1	1	1	1	1	1	1	1	1	1	1	1	1	1	1	1	2
**HRT**	1	1	1	1	1	1	1	1	1	1	1	1	1	1	1	1	38
**HSF**	0.844	1	0.286	0.275	0.766	1	0.255	0.170	0.626	1	0.065	0.660	0.239	0.271	0.437	1	3
**IWS1**	1	1	1	1	1	1	1	1	1	1	1	1	1	1	1	1	25
**Jumonji**	1	1	1	1	1	1	1	1	1	1	1	1	1	1	1	1	2
**LFY**	1	1	1	1	1	1	1	1	1	1	1	1	1	1	1	1	11
**LIM**	1	1	1	0.261	1	1	1	0.345	1	1	1	0.268	1	0.258	1	1	69
**LOB**	0.643	1	0.861	0.852	0.114	1	0.533	0.931	0.530	0.720	0.944	0.012	1	0.557	0.756	1	6
**LUG**	1	1	1	1	1	1	1	1	1	1	1	1	1	1	1	1	97
**MADS**	0.950	0.152	1	0.492	0.711	0.741	0.922	0.887	0.974	0.257	1	0.512	0.427	0.740	0.523	0.554	4
**MBF1**	1	1	1	1	1	1	1	1	1	1	1	1	1	1	1	1	2
**MED6**	1	1	1	1	1	1	1	1	1	1	1	1	1	1	1	1	2
**MED7**	1	1	1	1	1	1	1	1	1	1	1	1	1	1	1	1	30
**mTERF**	0.768	0.065	1	0.194	1	1	0.543	1	0.497	0.102	0.344	1	0.167	1	0.325	1	193
**MYB**	0.009	1	0.041	0.265	0.005	0.496	0.025	0.028	0.106	0.973	0.155	0.291	0.193	0.596	0.003	0.502	128
**MYB-related**	0.948	0.538	0.701	0.681	0.526	0.833	0.418	0.958	0.562	0.201	0.770	0.285	0.204	0.450	0.377	0.706	158
**NAC**	0.035	0.897	0.072	0.933	0.002	1	0.002	0.985	0.002	0.948	0.001	0.452	0.038	0.417	0.001	0.077	55
**OFP**	0.744	0.541	1	0.441	1	0.172	1	0.343	0.590	0.637	0.659	0.457	1	0.015	0.636	0.085	146
**Orphans**	0.700	0.333	0.587	0.563	0.976	0.003	0.979	0.473	0.701	0.498	0.236	0.985	0.147	0.909	0.827	0.141	3
**PBF-2-like**	1	1	1	1	1	1	1	1	1	1	1	1	1	1	1	1	61
**PHD**	1	1	1	1	1	1	1	1	1	1	1	1	1	1	1	1	21
**PLATZ**	0.262	1	0.449	1	0.551	1	1	0.554	0.696	1	0.581	1	1	1	1	0.332	3
**Pseudo ARR-B**	1	1	1	1	1	1	1	1	1	1	1	1	1	1	1	1	7
**RB**	1	1	1	1	1	1	1	1	1	1	1	1	1	1	1	1	3
**Rcd1-like**	1	1	1	1	1	1	1	1	1	1	1	1	1	1	1	1	22
**RWP-RK**	1	1	1	1	0.568	1	0.436	1	1	0.332	1	1	1	1	1	0.064	2
**S1Fa-like**	0.093	1	1	1	0.073	1	1	1	0.107	1	1	1	1	1	0.076	1	38
**SBP**	0.844	1	1	1	0.766	1	1	0.769	0.884	1	1	0.660	1	1	0.437	1	57
**SET**	1	1	1	1	1	1	1	1	1	1	1	1	1	1	1	1	10
**Sigma70-like**	1	1	1	1	1	1	1	1	1	0.167	1	1	1	1	1	1	41
**SNF2**	1	1	1	1	1	1	1	1	1	1	1	1	1	1	1	1	1
**SOH1**	1	1	1	1	1	1	1	1	1	1	1	1	1	1	1	1	15
**SRS**	1	1	1	0.338	1	1	1	1	1	1	1	0.346	0.312	1	1	1	25
**SWI/SNF-BAF60b**	1	1	1	1	1	1	1	1	1	1	1	1	1	1	1	1	5
**SWI/SNF-SWI3**	1	1	1	1	1	1	1	1	1	1	1	1	1	1	1	1	8
**TAZ**	1	1	1	1	1	1	1	1	1	1	1	1	1	1	1	1	54
**TCP**	0.929	0.534	0.785	1	1	0.527	0.406	0.015	0.954	0.256	1	0.448	0.741	0.427	1	0.647	50
**Tify**	0.913	0.507	0.409	0.152	0.564	1	0.136	0.568	0.137	0.602	0.050	0.759	0.714	0.389	0.306	1	51
**TRAF**	1	1	1	1	1	1	1	1	1	1	1	1	1	1	1	1	44
**Trihelix**	0.153	1	0.123	1	1	0.456	0.683	0.229	0.706	1	0.537	1	0.294	0.330	0.826	0.203	21
**TUB**	0.262	1	0.449	1	1	1	1	1	0.696	1	1	1	1	1	1	1	3
**ULT**	1	1	1	0.079	0.108	1	1	1	1	1	1	0.081	1	1	0.112	1	5
**VOZ**	1	1	1	1	1	1	1	1	1	1	1	1	1	1	1	1	160
**WRKY**	0.346	0.662	0.076	0.636	0.002	0.895	0.002	0.862	0.000	0.950	0.000	0.943	0.761	0.628	0.594	0.592	23
**zf-HD**	0.674	1	1	1	0.585	1	1	0.214	0.363	1	0.614	1	1	1	0.598	1	60
