# Supplementary material for: Genetic and Molecular Characterization of Submergence Response Identifies Subtol6 as a Major Submergence Tolerance Locus in Maize
Source: PLoS One. 2015 Mar 25;10(3):e0120385. doi: 10.1371/journal.pone.0120385 (PMC4373911; doi:10.1371/journal.pone.0120385)
Supplement: S3 Table — (PDF) [file pone.0120385.s014.pdf]

S3 Table. Summary of comparative analysis of maize, Arabidopsis and rice submergence transcriptome analysis.

	24h				72h				Arabidopsis Submergence (GSE24077)				Rice Submergence											
	B73	B97	Mo18w	M162w	B73	B97	Mo18w	M162w	7hr Sub/Cont	7hr pvalue	24h Sub/Cont	24h pvalue	M202 0d/ M202 1d	M202 pvalue	Sub1A 0d/ Sub1A 1d	Sub1A pvalue	M202 1d/ Sub1A 1d	M202 6d/ Sub1A 6d	Rice Locus	Rice Affymetrix ProbeSet	Maize Annotation	TF Family	Arabidopsis Annotation	
GRMZM2G137341	-	-	8.2	3.4	3.6	-	-	3	-0.1	7.89E-01	0	9.85E-01	-0.5	9.01E-02	-0.4	2.61E-01	-0.01	1.87	LOC_Os02g45450	Os.51078.1.S1_at	dehydration-responsive element-binding protein 1A	ERF (A-1)	AT4G25470	CBF2
GRMZM2G175856	6.8	-	-	-	7.6	4.1	-	3.9	-0.2	1.58E-01	0.1	5.96E-01	-0.4	2.44E-01	-0.3	1.23E-02	0	0.01	LOC_Os08g43200	OsAffx.6121.1.S1_at	dehydration-responsive element-binding protein 1B	ERF (A-1)	AT5G51990	CBF4
GRMZM2G042756	3.6	2.2	6.2	3.6	5.6	-	4.6	2.4	-0.1	7.89E-01	0	9.85E-01	-0.1	8.67E-01	-0.7	1.07E-01	-0.07	1.33	LOC_Os04g48350	Os.57527.1.S1_at	dehydration-responsive element-binding protein 1D	ERF (A-1)	AT4G25470	CBF2
GRMZM2G124011	3.9	4.7	5.9	4	3.7	3.6	5.3	3.9	-0.3	5.46E-02	-0.1	4.03E-01	0.7	3.76E-02	0.7	1.76E-02	0.26	1.29	LOC_Os09g35030	Os.14125.1.S1_at	sbCBF6	ERF (A-1)	AT4G25480	CBF3
GRMZM2G069146	2.1	-	3.7	3.3	2.1	-	2.7	2	-0.1	7.89E-01	0	9.85E-01	0.7	3.76E-02	0.7	1.76E-02	0.26	1.29	LOC_Os09g35030	Os.14125.1.S1_at	DNA binding	ERF (A-1)	AT4G25470	CBF2
GRMZM2G069126	-	-	4.3	3.5	-	-	2.5	-	-0.1	7.89E-01	0	9.85E-01	0.7	3.76E-02	0.7	1.76E-02	0.26	1.29	LOC_Os09g35030	Os.14125.1.S1_at	sbCBF6	ERF (A-1)	AT4G25470	CBF2
GRMZM2G174917	1.6	-	2.3	-	1.7	1.3	2.5	1.4	-2.5	5.39E-08	-1.9	1.24E-06	0	4.96E-01	0.5	2.73E-02	-0.09	0.5	LOC_Os04g55520	Os.22629.1.S1_at	dehydration responsive element binding protein	ERF (A-5)	AT2G23340	DEAR3
GRMZM2G068967	3.6	2.8	4.2	3.4	3.3	2.2	2.6	3.4	0.6	1.15E-03	1	5.32E-06	1.4	1.39E-03	3	1.62E-02	0.05	0.45	LOC_Os04g52090	OsAffx.14373.1.S1_s_at	ethylene-responsive transcription factor 4	ERF (B-1)	AT3G20310	ERF7
GRMZM2G020054	4	2.6	5.5	3.8	3.4	1.9	2.9	3.3	0.6	1.15E-03	1	5.32E-06	1.4	1.39E-03	3	1.62E-02	0.05	0.45	LOC_Os04g52090	OsAffx.14373.1.S1_s_at	DNA binding	ERF (B-1)	AT3G20310	ERF7
GRMZM2G138396	-	-	7	4.7	5.8	-	-	-	-2.1	1.88E-02	-2.1	1.79E-02	0.1	7.45E-01	-0.6	1.58E-01	-0.94	2.13	LOC_Os09g28440	Os.52451.1.A1_at	DNA binding	ERF (B-2)	AT4G34410	RRTF1
GRMZM2G081892	1.1	1.6	1.5	-	-	-	-	1.1	-	-	-	-	1.2	6.29E-04	1.1	2.63E-02	0.3	0.08	LOC_Os01g04020	Os.4663.1.S1_at	pathogenesis-related transcriptional activator PTI6	ERF (B-2)	AT1G68550	-
GRMZM2G131281	-	1.9	-	-	2.8	2.8	-	2.1	0.2	1.39E-01	0.4	1.57E-02	1.2	2.33E-02	1.3	1.12E-02	0.07	-0.1	LOC_Os09g26420	Os.5850.1.S1_at	DNA binding	ERF (B-2)	AT5G61890	-
GRMZM2G148333	3	3.4	3.8	3.6	3.3	3.4	3.5	3.2	4.1	2.59E-11	3.9	4.27E-11	1.2	2.33E-02	1.3	1.12E-02	0.07	-0.1	LOC_Os09g26420	Os.5850.1.S1_at	ethylene response factor	ERF (B-2)	AT1G72360	HRE1
GRMZM2G052667	3	3.6	4.3	3.5	2.7	3.4	4.4	3.2	4.1	2.59E-11	3.9	4.27E-11	1.2	2.33E-02	1.3	1.12E-02	0.07	-0.1	LOC_Os09g26420	Os.5850.1.S1_at	DNA binding	ERF (B-2)	AT1G72360	HRE1
GRMZM2G085964	3.6	-	5	4.1	5.2	5.2	7	5.8	4.1	9.18E-14	4.8	1.49E-14	4.2	1.39E-03	4.5	3.07E-03	0.6	1.59	LOC_Os05g29810	Os.4893.1.S1_at	DNA binding	ERF (B-2)	AT3G16770	ERF72
GRMZM2G018984	4.4	7	8.7	8.2	5.3	7	8.7	8.1	1.8	3.50E-08	2.1	6.95E-09	4.8	2.20E-05	4.7	6.28E-04	0.16	0.59	LOC_Os03g08470	Os.6009.3.S1_a_at	hypothetical protein LOC100192457	ERF (B-2)	AT1G53910	RAP2.12
GRMZM2G169382	3.4	-	2.5	3.5	4.9	-	3.8	4.9	0.9	2.64E-02	0.3	5.29E-01	5.5	2.23E-03	5.8	5.28E-04	0.48	2	LOC_Os01g21120	Os.8031.1.S1_at	hypothetical protein LOC100193143	ERF (B-2)	AT2G47520	HRE2
GRMZM2G129674	11.8	3.9	8.6	8.4	13.7	4.9	1.8	1.4	4.1	2.59E-11	3.9	4.27E-11	6.5	2.04E-03	6.8	3.58E-03	0.12	0.61	LOC_Os03g08460	Os.11120.1.S1_at	LOC100216626	ERF (B-2)	AT1G72360	HRE1
GRMZM2G053503	5.5	3.4	4.8	5.9	6.2	4.1	5.3	6.9	-	-	-	-	5.5	2.23E-03	5.8	5.28E-04	0.48	2	LOC_Os01g21120	Os.8031.1.S1_at	ethylene-responsive factor-like protein 1	ERF (B-2)	-	-
GRMZM2G159592	3.6	5.1	8	6.5	3.9	5.3	8.6	6.5	2	2.08E-08	2.5	9.61E-10	0.3	6.17E-01	-0.1	7.22E-01	0.37	-0.01	LOC_Os05g47650	OsAffx.15137.1.S1_at	DNA binding	ERF (RAV3)	AT1G13260	RAV1
GRMZM2G169654	2.2	2.5	2.1	-	2.2	2.8	1.4	1.7	2.9	3.07E-11	3.6	1.60E-12	0.7	3.05E-02	1	8.10E-05	0.25	0.54	LOC_Os01g49830	Os.8019.1.S1_at	DNA-binding protein RAV1	ERF (RAV3)	AT1G68840	RAV2
GRMZM2G059939	2.7	3	2.3	2.9	2.5	2.8	1.8	2.9	2	2.08E-08	2.5	9.61E-10	3.5	4.23E-04	3.2	6.12E-04	0.23	-0.32	LOC_Os05g47650	OsAffx.15137.1.S1_at	DNA binding	ERF (RAV3)	AT1G13260	RAV1
GRMZM2G038821	1.7	-	3.9	-	-	-	-	-	2	2.52E-08	0.8	1.34E-03	0.03	9.58E-01	0.32	9.48E-02	-0.1	-1.13	LOC_Os01g06660	Os.1445.1.S1_at	pyruvate decarboxylase		AT5G54960	PDC2
GRMZM2G073044	2.2	3.9	5	4.7	3.1	3.1	5.5	5	4.6	4.27E-12	4.5	5.90E-12	3.49	2.01E-02	3.6	2.07E-02	0.45	1.54	LOC_Os01g32770	Os.22577.1.S1_x_at	seed specific protein Bn15D17A	LOB	AT3G02550	LBD41
GRMZM2G386674	3.2	3.9	5.1	6.6	4.6	4.1	5.8	7.3	4.6	4.27E-12	4.5	5.90E-12	3.49	2.01E-02	3.6	2.07E-02	0.45	1.54	LOC_Os01g32770	Os.22577.1.S1_x_at	seed specific protein Bn15D17A	LOB	AT3G02550	LBD41
GRMZM2G305362	7	7.9	8.7	10.4	9	8.2	1.5	13.1	2	4.20E-06	0.7	4.57E-02	-0.59	2.59E-01	-0.73	7.30E-03	0.03	-0.18	LOC_Os12g06640	Os.10232.1.A1_at	Transcribed locus		AT3G10040	
GRMZM2G442658	3.2	1.9	4.8	5.1	3.8	2.8	5.5	5.8	3	3.05E-06	1.9	4.66E-04	2.87	6.03E-03	4.58	1.52E-03	1.24	1.86	LOC_Os11g10480	Os.12591.2.S1_x_at	zinc ion binding		AT1G77120	ADH
