# Supplementary material for: Genetic and Molecular Characterization of Submergence Response Identifies Subtol6 as a Major Submergence Tolerance Locus in Maize
Source: PLoS One. 2015 Mar 25;10(3):e0120385. doi: 10.1371/journal.pone.0120385 (PMC4373911; doi:10.1371/journal.pone.0120385)
Supplement: S4 Table — NA: not included in the classification by McMullen et al., [29]. (PDF) [file pone.0120385.s015.pdf]

S4 Table. Maize varieties and subfamily classification (29). NA: not included in the classification by McMullen et al., (29).

Variety	Subfamily
B73	NA
B97	NSS
CML103	Tropical
CML228	Tropical
CML247	Tropical
CML277	Tropical
CML322	Tropical
CML333	Tropical
CML52	Tropical
CML69	Tropical
HP301	Other
Il14H	Other
Ki11	Tropical
Ky21	NSS
M162W	NSS
Mo18W	Mixed
Ms71	NSS
NC350	Tropical
NC358	Tropical
Oh43	NSS
Oh7B	NSS
Tx303	Mixed
Tzi8	Tropical
