# Supplementary material for: Genetic and Molecular Characterization of Submergence Response Identifies Subtol6 as a Major Submergence Tolerance Locus in Maize
Source: PLoS One. 2015 Mar 25;10(3):e0120385. doi: 10.1371/journal.pone.0120385 (PMC4373911; doi:10.1371/journal.pone.0120385)
Supplement: S5 Table — Mapping was done using Bowtie and Tophat as described in Materials and Methods. (PDF) [file pone.0120385.s016.pdf]

S5 Table. Detailed mapping information for all 36 RNA-seq samples. Mapping was done using Bowtie and Tophat as described in Materials and Methods.

Inbred	Time	Treatment	Rep	Total reads	Mapped reads	Unique reads
B97	72h	Submerged	1	40602519	22998687	20876031
B97	72h	Submerged	2	33274356	18297372	16388723
B97	72h	Submerged	3	30873281	17689088	15524797
Mo18W	72h	Submerged	1	31702648	17170552	15021700
Mo18W	72h	Submerged	2	32404534	17807409	15990448
Mo18W	72h	Submerged	3	33681068	18314970	16582453
M162W	72h	Submerged	1	27831500	15866717	14046889
M162W	72h	Submerged	2	30974798	17118949	15107963
M162W	72h	Submerged	3	35891773	20280691	18109335
B73	24h	Control	1	28369161	16496388	15118808
B73	24h	Control	2	29321780	17264287	15833521
B73	24h	Control	3	25237690	14991060	13726624
B97	24h	Control	1	31356907	16632472	15020298
B97	24h	Control	2	32799769	17099857	15542828
B97	24h	Control	3	31162018	16525246	14949959
Mo18W	24h	Control	1	28548359	16349081	14407921
Mo18W	24h	Control	2	25760142	13138623	11907877
Mo18W	24h	Control	3	45738357	24728759	22455582
M162W	24h	Control	1	42304662	23260783	20888814
M162W	24h	Control	2	35832663	19384400	17515357
M162W	24h	Control	3	34535719	18574325	16927615
B73	24h	Submerged	1	38823529	27468609	19992084
B73	24h	Submerged	2	35676878	23725752	21438790
B73	24h	Submerged	3	30137388	19331317	17314796
B97	24h	Submerged	1	42587599	24899452	21808426
B97	24h	Submerged	2	39195745	22691399	19775623
B97	24h	Submerged	3	44334211	25773067	22659936
Mo18W	24h	Submerged	1	40009294	22842495	19644268
Mo18W	24h	Submerged	2	39929607	22151346	19565898
Mo18W	24h	Submerged	3	33810057	18289131	16177832
M162W	24h	Submerged	1	30318702	16992094	14750814
M162W	24h	Submerged	2	34171144	18725679	16755845
M162W	24h	Submerged	3	38885567	21931110	19570034
B73	72h	Submerged	1	26873974	17741634	16180818
B73	72h	Submerged	2	29567026	19397347	17715062
B73	72h	Submerged	3	45187585	29952601	27492261
**Average**				34380889	19775076	17577390
**Standard Deviation**				5644558.0	3773029.7	3192561.2
