# Supplementary material for: Genetic and Molecular Characterization of Submergence Response Identifies Subtol6 as a Major Submergence Tolerance Locus in Maize
Source: PLoS One. 2015 Mar 25;10(3):e0120385. doi: 10.1371/journal.pone.0120385 (PMC4373911; doi:10.1371/journal.pone.0120385)
Supplement: S6 Table — (PDF) [file pone.0120385.s017.pdf]

S6 Table. List of Primers

Gene ID	Gene Name	Forward Primer	Right Primer	Product Size	Notes
GRMZM2G442658	*Alcohol Dehydrogenase (ADH)*	TAACTAACTCGTTGAGTGGCC	CTCCTCGATCGACAGTGGCT	197	-
GRMZM2G087186	*Pyruvate Decarboxylase (PDC)*	ACCGAACCAAACAACACACGTC	GCCAACCGTCTGGTTCGA	210	-
TCONS_00005048	*Pyrophosphate-Dependent Fructose-6-Phosphate 1-Phosphotransferase (PFP)*	GCGATTATGGCTACGTTCTTGG	CCATGTGGACAGCAGGCTTA	202	-
GRMZM2G125669	*Alternative Oxidase 1a (AOX1a)*	AGTCGCTGCGCTTCCCCA	CGACGCGGTGCGCGAACT	324	ROS Marker
GRMZM2G087875	*CYP81D8*	GTGCGGCCAACCTGTGGA	GTTAAGCAAGAGCGACATTGC	341	ROS Marker
GRMZM5G871347	*WRKY6*	ATCCGTCGACAACAGCAAGG	CCTCGTAGGTAGTGATGAGGAT	258	ROS Marker
GRMZM2G102471	*Ubiquitin-Conjugating Enzyme*	GCTAAAAGCTGGCCAAACTG	AACAGCAACACCACAAACCA	243	qPCR Internal Control
GRMZM2G059939	*RELATED TO ABA-INSENSITIVE3(ABI3)/ VIVIPAROUS1 (RAV1)*	GTACTCGTACTGGAACAGCAG	GTGGTGGTCATCGCGTTG	210	-
GRMZM2G168898	*HEMOGLOBIN2 (HB2)*	GTTCAGTGAGGCACAGGAAG	GCGCAGGAAGGAGAACATC	139	-
